# Dual‐Strategy Direct Photocatalytic Patterning for Efficient Perovskite Nanocrystal LED Displays

**DOI:** 10.1002/adma.202508217

**Published:** 2025-07-31

**Authors:** Seongkyu Maeng, Junho Kim, Taehyun Kim, Seyun Lee, Seunghee Han, Sun Jae Park, Changjo Kim, Jihan Kim, Jung‐Yong Lee, Himchan Cho

**Affiliations:** ^1^ Department of Materials Science and Engineering Korea Advanced Institute of Science and Technology (KAIST) 291, Daehak‐ro Yuseong‐gu Daejeon 34141 Republic of Korea; ^2^ School of Electrical Engineering Korea Advanced Institute of Science and Technology (KAIST) 291, Daehak‐ro Yuseong‐gu Daejeon 34141 Republic of Korea; ^3^ Graduate School of Semiconductor Technology Korea Advanced Institute of Science and Technology (KAIST) 291, Daehak‐ro Yuseong‐gu Daejeon 34141 Republic of Korea; ^4^ Department of Chemical and Biomolecular Engineering Korea Advanced Institute of Science and Technology (KAIST) 291, Daehak‐ro Yuseong‐gu Daejeon 34141 Republic of Korea

**Keywords:** direct optical patterning, electroluminescence, film‐state ligand exchange, ligand crosslinking, metal halide perovskites

## Abstract

Achieving nondestructive, high‐resolution patterning of perovskite nanocrystals (PeNCs) is essential for next‐generation near‐eye displays. However, the intrinsic instability of PeNCs renders conventional patterning methods detrimental to their optical and electrical properties. Herein, a dual strategy is reported that enables both high‐resolution patterning and the fabrication of efficient light‐emitting diodes (LEDs). The first strategy involves an advanced direct photocatalytic patterning method. Thiol crosslinkers are systematically investigated and identified 1,8‐octanedithiol and 1,10‐decanedithiol as optimal candidates due to their solvent compatibility, colloidal stability, and ability to achieve nondestructive patterning with high resolution and fidelity. The second strategy introduces a film‐state ligand exchange (FLE) process to enhance the optical and electrical properties of patterned PeNC films. Replacing long‐chain ligands with short‐chain ammonium halides results in denser surface passivation and enhanced charge transport capability. Dual strategy enabled high‐performance crosslinked PeNC‐LEDs, including a maximum external quantum efficiency of 14.7% and luminance of ≈25,400 cd m^−2^ for green CsPbBr_3_ LEDs, representing the highest values reported for green CsPbBr_3_ PeNC‐LEDs obtained via direct optical patterning. Furthermore, FLE enabled post‐patterning halide exchange, representing the first demonstration of a red crosslinked CsPbBr_x_I_3−x_ PeNC‐LED via direct optical patterning. This study establishes molecular and lithographic design principles for integrating colloidal nanocrystals into next‐generation displays and optoelectronics.

## Graphical abstract

A dual‐strategy is presented direct photocatalytic patterning that enables high‐resolution, nondestructive patterning and fabrication of efficient crosslinked perovskite nanocrystal‐light‐emitting diodes (PeNC‐LEDs). The first strategy systematically identifies thiol crosslinkers for advanced direct photocatalytic patterning. The second applies film‐state ligand exchange to enhance optical and electrical properties. Dual strategy demonstrates CsPbBr_3_ PeNC‐LEDs with 14.7% external quantum efficiency and ≈25,400 cd m^−2^ luminance after patterning process.

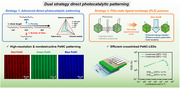

## 1 Introduction

Perovskite nanocrystals (PeNCs) have garnered significant attention as next‐generation colloidal emissive nanomaterials for various optoelectronic devices owing to their high photoluminescence quantum yield (PLQY), narrow emission spectra, defect tolerance, facile color tunability, and solution processability.^[^ ^1^, ^2^, ^3^ ^]^ Their superior color purity compared to other emissive nanomaterials, such as quantum dots (QDs) and organic emitters, enables broad coverage of the Rec.2020 color space, making them ideal for future display applications.^[^ ^4^ ^]^ In photoluminescence (PL) applications, PeNCs exhibit a high color conversion efficiency owing to their high PLQY and molar extinction coefficient.^[^ ^5^, ^6^ ^]^ In electroluminescence (EL) applications, PeNCs were demonstrated to achieve high external quantum efficiencies (EQEs) exceeding 20% when used as the emissive layer in light‐emitting diodes (LEDs).^[^ ^7^, ^8^ ^]^ Hence, the next step in their advancement involves developing high‐resolution pixel patterning technologies, which are crucial for incorporating these materials into next‐generation displays. Recent advancements demand resolutions exceeding several thousand pixels per inch (PPI) for near‐eye displays in augmented reality (AR) and virtual reality (VR).^[^ ^9^, ^10^, ^11^, ^12^, ^13^ ^]^ However, conventional patterning techniques, such as inkjet printing,^[^ ^14^, ^15^, ^16^, ^17^ ^]^ transfer printing,^[^ ^18^, ^19^, ^20^ ^]^ and photolithography,^[^ ^21^ ^]^ struggle to produce high‐resolution, high‐uniformity PeNC patterns with high throughput.

Direct optical patterning is an emerging, alternative patterning technique for QDs and PeNCs that utilizes solubility changes triggered by photo‐induced chemical reactions using photo‐sensitive ligands or additives. This approach eliminates the need for conventional polymeric photoresist, enabling direct patterning of nanomaterials. It thus facilitates the fabrication of high‐resolution patterns through a simple process and offers high fidelity and throughput, which has led to extensive research on various photo‐induced chemical reactions.^[^ ^9^, ^22^, ^23^, ^24^, ^25^, ^26^, ^27^, ^28^, ^29^, ^30^, ^31^, ^32^, ^33^, ^34^, ^35^, ^36^, ^37^ ^]^ However, during the ultraviolet (UV) exposure step, inorganic nanomaterials can degrade due to light‐induced degradation, and radicals generated from photo‐sensitive molecules form surface traps.^[^ ^25^, ^26^, ^29^, ^38^, ^39^ ^]^ Therefore, various strategies have been introduced to achieve non‐destructive QD‐LED fabrication after the patterning process. One effective approach is increasing the shell thickness of II–VI QDs to protect the emissive core, thereby enabling pattern formation without compromising optical properties.^[^ ^38^ ^]^ Alternatively, modifying the structure of photo‐sensitive molecules to prevent the formation of radicals on the surface or modulating the UV wavelength can help alleviate the formation of surface traps in QDs.^[^ ^26^, ^40^ ^]^ Through these strategies, the PLQY of II–VI and III–V QDs can be maintained at over 90% of their initial value after the direct optical patterning process. Beyond improvements in optical properties, optimizations aimed at enhancing electrical properties have recently resulted in QD‐LEDs attaining EQEs approaching 20% even after the patterning process.^[^ ^26^, ^33^, ^36^ ^]^

However, PeNCs typically lack a shell and have an ionic nature, rendering them highly susceptible to detrimental effects from the environment and reactive additives.^[^ ^41^ ^]^ Consequently, there is a significant reduction in the PLQY during the PeNC patterning process.^[^ ^25^, ^29^, ^32^, ^37^ ^]^ The electrical properties of PeNC‐LEDs can also substantially deteriorate after the patterning process. In particular, LEDs based on CsPbBr_3_ PeNCs after the patterning process have shown *EQE_max_* below 2% and maximum luminance (*L_max_*) under 2000 cd m^−2^.^[^ ^25^, ^42^, ^43^ ^]^ Post‐ligand treatments have been applied to recover the degraded optical properties; however, while this approach can passivate defects, it is ineffective in improving electrical properties when utilizing long‐chain ligands, as they can hinder charge injection and transport in LEDs.^[^ ^25^, ^27^, ^32^ ^]^ This necessitates the development of strategies that can improve both optical and electrical properties of patterned PeNCs.

To achieve optically nondestructive PeNC patterning, our group recently proposed an advanced direct optical patterning strategy, named direct photocatalytic patterning, which integrates the photocatalytic activity of emissive nanomaterials with the direct optical patterning process.^[^ ^37^ ^]^ By utilizing pentaerythritol tetrakis(3‐mercaptopropionate) (PTMP), a molecule with four thiol end‐groups, high‐resolution patterns were fabricated through a thiol‐ene reaction without any degradation in the optical properties.^[^ ^37^ ^]^ The photocatalytic activity of PeNCs significantly improves the generation of thiyl radicals, enabling the activation of the thiol‐ene reaction with a low UV dose. However, this approach can be effectively applied only to PeNCs with oleylammonium ligands, rendering its extension to other ligand systems challenging. Furthermore, owing to the insulating properties imparted by the long aliphatic chains of the oleylammonium ligands, crosslinked CsPbBr_3_ PeNC‐LEDs exhibited a low *EQE_max_* of ≈2.2% and a *L_max_* of ≈450 cd m^−2^, falling short of industrial requirements.

In this work, we introduce a dual‐strategy direct photocatalytic patterning method that enables both high‐resolution patterning and the fabrication of efficient PeNC‐LEDs. As the first strategy, we build upon our previous direct photocatalytic patterning method, advancing it through a systematic study of thiol crosslinkers tailored for ligand crosslinking and compatibility with solution‐processed device fabrication. We utilize 1,8‐octanedithiol (ODT) and 1,10‐decanedithiol (DDT), which exhibit wide solvent compatibility, high colloidal stability in formulated PeNC inks, and excellent patternability. To further enhance charge transport properties and passivate surface defects in the crosslinked state, we introduce a film‐state ligand exchange (FLE) approach as the second approach. The FLE effectively modifies the surface ligands of crosslinked PeNCs, thereby enhancing their optical and electrical properties. By leveraging this dual‐strategy approach, we achieved efficient crosslinked green CsPbBr_3_ PeNC‐LEDs, demonstrating its applicability in enabling high‐resolution patterning without compromising EL performance. FLE also enables precise control over the halide composition of crosslinked PeNCs, facilitating the tunability of the emission wavelength in the film state. By further developing this approach, we achieved the first successful demonstration of red crosslinked CsPbBr_x_I_3−x_ PeNC‐LEDs prepared using the direct optical patterning process. Our dual strategy provides a technological roadmap in the material and process design required for nondestructive direct optical patterning, which can help significantly advance the application of colloidal nanocrystals in optoelectronic devices.

## 2 Results and Discussion

To achieve high‐efficiency PeNC‐LEDs with photopatternable PeNC inks (i.e., compatible with a direct photocatalytic patterning process), the first step is to develop a widely applicable thiol crosslinker that enables nondestructive patterning. However, previous studies using PTMP have encountered issues with ligand detachment when applied to PeNCs synthesized without excess halide sources, resulting in a significant reduction in PLQY upon patterning.^[^ ^37^ ^]^ To address this issue, we identified a thiol crosslinker that is broadly applicable to colloidal PeNC solutions without negatively affecting the LED fabrication process.

The thiol crosslinker for advanced direct photocatalytic patterning must meet three criteria: solvent compatibility, colloidal stability, and patternability. First, the crosslinker must be soluble in the solvent used to disperse the material for patterning. For instance, a bis‐azide crosslinker was modified by introducing a hydrophilic unit, rendering it alcohol‐soluble.^[^ ^44^ ^]^ Second, in the fabrication of solution‐processed LEDs, the selection of an appropriate solvent is crucial to minimizing its impact on other layers, particularly the charge transport layer; in other words, orthogonal processing is necessary. In previous studies, a bis‐azide crosslinker, insoluble in octane but soluble in toluene, necessitated the use of toluene as the solvent in the PeNC ink formulation for LED fabrication.^[^ ^29^ ^]^ However, toluene can partially dissolve underlying polymeric hole transport layers (e.g., Poly(N,N“‐bis‐4‐butylphenyl‐N,N”‐bisphenyl)benzidine (Poly‐TPD), poly(9‐vinylcarbazole) (PVK), and poly(triaryl amine) (PTAA)), significantly reducing device efficiency.^[^ ^29^ ^]^ Therefore, to ensure orthogonal processing, a crosslinker with high solubility across a range of solvents is required. Moreover, the formulated photopatternable inks prepared by mixing the crosslinker molecules with PeNCs must exhibit a high colloidal stability. Nanocrystal aggregation during the formulation step makes it difficult to achieve well‐defined patterns. Finally, the crosslinker must enable efficient crosslinking with a minimal UV dose, inducing solubility changes that allow the fabrication of high‐resolution patterns while minimizing UV‐induced material degradation.

**Figure 1:**
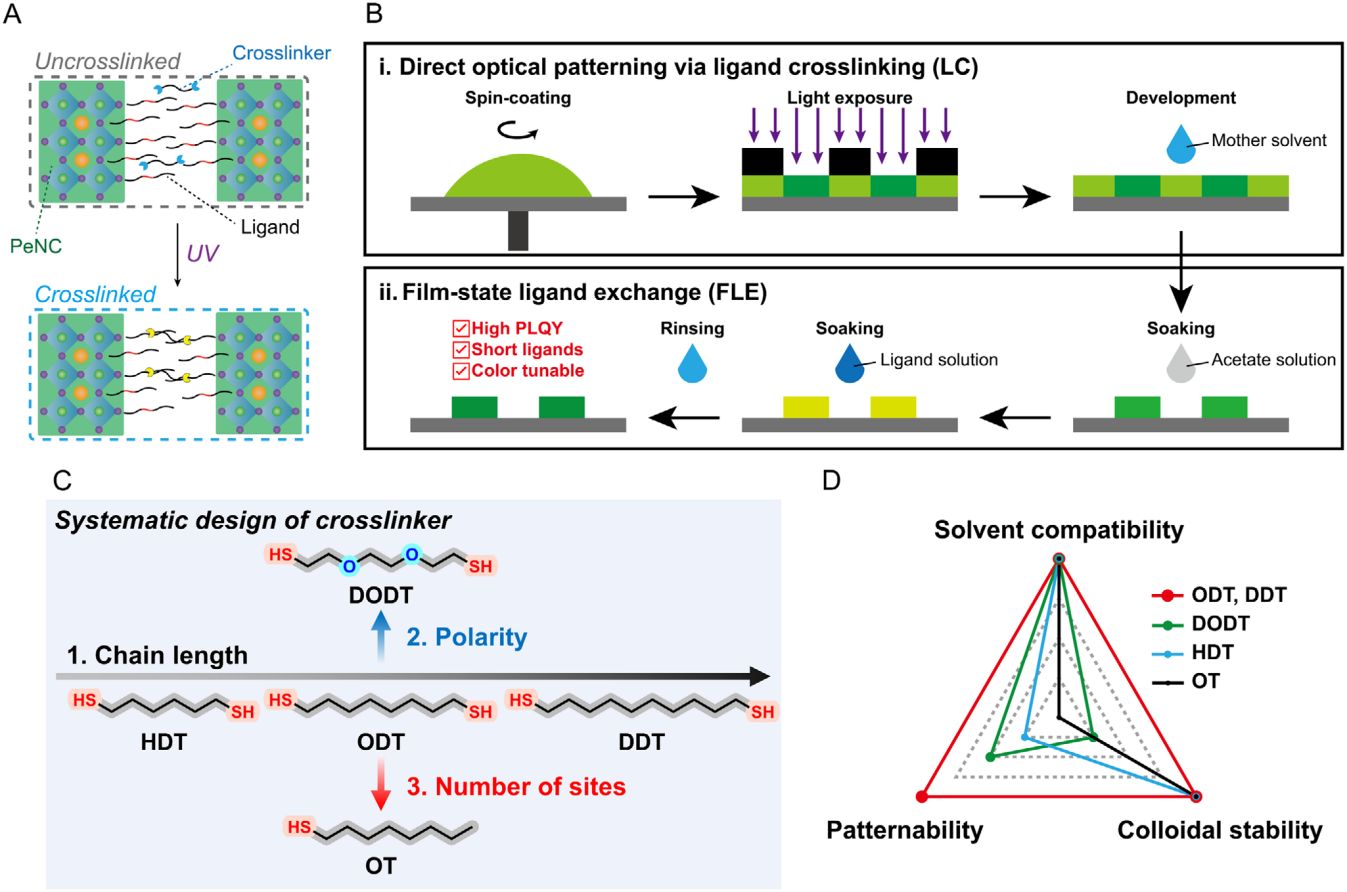
Schematics of the nondestructive direct photocatalytic patterning process: A) Schematic of the direct photocatalytic patterning mechanism. B) Schematic of the direct optical patterning via ligand crosslinking (LC) and film‐state ligand exchange (FLE). C) Chemical structures of thiol crosslinker molecules utilized for direct photocatalytic patterning. D) Radar chart illustrating the key parameters of the thiol crosslinker molecules involved in direct photocatalytic patterning.

**Figure** **1**A shows a scheme of the ligand crosslinking (LC) mechanism. Crosslinking between the PeNC ligands and the crosslinker under UV exposure leads to a change in the solubility. The photocatalytic activity of PeNCs promotes thiol‐ene reaction‐based LC, thereby facilitating pattern formation even with a low UV dose (Figure S1, Supporting Information).^[^ ^37^, ^45^, ^46^, ^47^ ^]^ Figure 1B‐i illustrates a schematic of the direct optical patterning process for the fabrication of PeNC patterns. 1) A PeNC thin film is fabricated by spin‐coating the formulated ink onto substrates such as glass, Si, or flexible substrates. 2) A pre‐made photomask is aligned with the PeNC thin film; the structure is then subjected to UV irradiation. 3) The unexposed areas are removed by development using a mother solvent, resulting in the formation of a pattern. Figure 1B‐ii shows the FLE, which includes: 1) soaking the PeNC film in an acetate solution to remove long‐chain ligands, 2) soaking the PeNC film in a ligand solution for ligand exchange and additional passivation, and 3) rinsing unbound ligands with a mother solvent. Further details of the dual strategy are described in the experimental section.

Figure 1C illustrates the systematic design of the thiol crosslinkers for advanced direct photocatalytic patterning. To identify the optimal molecule, we systematically adjusted the chain length, polarity, and the number of functional groups. The selected candidate molecules included 1,6‐hexanedithiol (HDT), ODT, and (DDT, which have two thiol functional groups and exhibit lower polarity as the alkyl chain length increases; 3,6‐dioxa‐1,8‐octanedithiol (DODT), which has two thiol functional groups and high polarity; and 1‐octanethiol (OT), which has a single thiol functional group.^[^ ^48^ ^]^ A systematic analysis revealed that ODT and DDT were the most suitable crosslinkers, as they demonstrated the ability to prepare formulated inks with high colloidal stability and effectively facilitated LC formation (Figure 1D).

The CsPbBr_3_ PeNCs used for patterning were synthesized via a hot injection method using oleic acid (OA) and oleylamine (OLA) as ligands (See Experimental Method in Supporting Information for further details). A transmission electron microscopy (TEM) analysis confirmed an average size of 11.1 nm (Figure S2, Supporting Information).^[^ ^49^ ^]^ We chose octane as the solvent for pristine PeNC dispersions, as toluene can dissolve the pre‐deposited hole transport layers during LED fabrication process.^[^ ^33^ ^]^

As a first step, the solubility of the thiol crosslinkers in octane was examined. PTMP, which was used in our previous study^[^ ^37^ ^]^ and a similar molecule with three thiol end‐group, trimethylolpropane tris(3‐mercaptopropionate) (TTMP), could not be utilized due to their high polarity, which prevents their dissolution in octane (Figure S3, Supporting Information).^[^ ^48^ ^]^ However, the other thiol crosslinkers (HDT, ODT, DDT, DODT, and OT) fully dissolved in octane at a concentration of 0.25 m. We then analyzed the colloidal stability of the formulated inks (Figure S4, Supporting Information). All the formulated inks exhibited high colloidal stability, except the DODT solution. The instability of DODT‐based PeNC inks arises from the higher polarity of DODT compared with other crosslinkers. These thiol crosslinkers can partially replace the initial oleate ligands through a protonation‐deprotonation equilibrium, where the thiol groups deprotonate to form thiolates (–S^−^). The presence of these thiolate ligands can increase the overall polarity of the PeNC surface, particularly for DODT, disrupting its dispersion in nonpolar solvents such as octane, even at low thiolate concentrations.^[^ ^50^, ^51^, ^52^ ^]^

**Figure 2:**
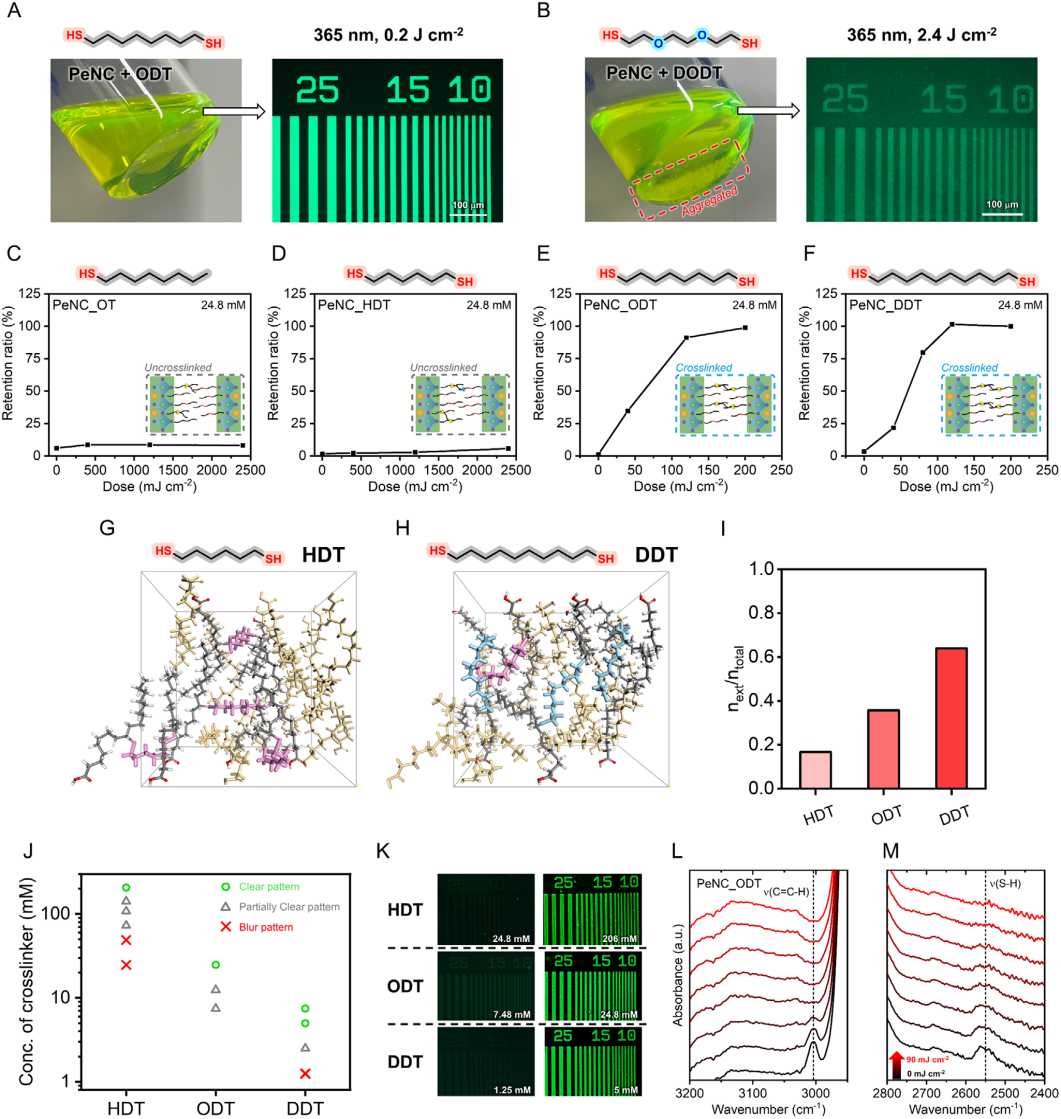
Chemical structure engineering of thiol crosslinkers for direct photocatalytic patterning: Formulation inks composed of PeNCs with A) ODT or B) DODT as the crosslinker in octane, and the corresponding PeNC patterns fabricated by the direct photocatalytic patterning process. (Concentration of the formulation ink: 22.7 mM) C‐F) Retention ratio measurements of PeNC films fabricated through direct photocatalytic patterning using OT, HDT, ODT, and DDT (all the samples with the same thiol molecule concentration of 24.8 mM). Representative conformations of crosslinking formed by G) HDT and H) DDT in MD simulations. Crosslinkers involved in internal crosslinking are shown in pink, while those involved in external crosslinking are shown in sky blue. Since the actual PeNC surface is larger, the x and y directions are assumed to be periodic. Ligands appearing outside the simulation space are shown to illustrate molecular connectivity and improve visual clarity. I) Average crosslinking efficiencies of different dithiol crosslinkers, obtained from MD simulations. J) Summary of F‐OM data under various thiol molecules (HDT, ODT, and DDT) and different thiol concentrations. “O” indicates a clear pattern with a high retention. “△” denotes a partially clear pattern with PL intensity between 1/3 and 2/3 of the maximum PL intensity. “X” represents a blur pattern with the PL intensity at or below 1/3 of the maximum PL intensity. K) F‐OM images of PeNC patterns fabricated using various thiol molecules (HDT, ODT, and DDT) and different thiol concentrations (all the samples exposed to a UV dose of 400 mJ cm^−2^). L‐M) FTIR spectra of the PeNC–ODT film under different UV exposure doses (0 to 90 mJ cm^−2^).

The colloidal stability of the formulated PeNC inks had a significant impact on pattern quality and the optimal UV dose. While ODT enabled the formation of high‐definition PeNC patterns with a UV dose of 0.2 J cm^−2^, the patterns created using DODT exhibited background residue and required a higher UV dose of 2.4 J cm^−2^ (UV wavelength = 365 nm for both), even after removing aggregated particles with a filtration step (**Figure** **2**A,B; Figure S5, Supporting Information).

To quantitatively determine the UV dose required for pattern fabrication, we measured the retention ratio for thiol crosslinkers. This ratio was defined as the absorbance at the first excitonic peak of the PeNC film after patterning, normalized to its initial absorbance before the process. Each thiol crosslinker was prepared at a consistent concentration of 24.8 mM in the formulated ink to determine the UV dose required for full retention (Figure 2C–F; Figure S6, Supporting Information). OT and HDT exhibited low film retention even at a high UV dose exceeding 2000 mJ cm^−2^, whereas ODT and DDT achieved full retention at doses above 120 mJ cm^−2^. The variation in retention ratios among different thiol crosslinkers can be understood as follows. OT, with a single thiol functional group, cannot induce crosslinking, thus failing to induce significant solubility contrast.^[^ ^53^ ^]^ Although HDT contains two thiol functional groups, its short chain length favors crosslinking within individual PeNCs rather than interparticle crosslinking, thereby hindering the generation of distinct solubility contrast required for patterning.^[^ ^40^, ^54^ ^]^ In contrast, ODT and DDT possess sufficiently long chains to enable interparticle crosslinking, enabling pattern formation with low UV doses. We performed molecular dynamics (MD) simulations to investigate the effect of crosslinker chain length on the crosslinking behavior (Figure 2G–I; Figure S7, Supporting Information). In the model, two parallel planes positioned along the top and bottom represented the surfaces of two different PeNCs, with only surface‐bound ligands included to simplify the system. To mimic the bound state, the terminal oxygen atom of each ligand was fixed in space. Dithiol crosslinkers were randomly distributed in the space between the two surfaces. To model bond formation, we assumed that an irreversible bond would form during the MD simulation whenever a sulfur atom from a crosslinker approached within a defined cutoff distance of a C═C bond in a surface ligand. This was implemented using a distance‐based bond formation algorithm, which has been previously applied to simulate self‐assembly processes (See Experimental Method in Supporting Information for further details).^[^ ^55^, ^56^ ^]^ After the simulations, we classified the total crosslinking (n_total_) into two types: interparticle crosslinking (or external crosslinking, n_ext_, shown in skyblue), which contributes to solubility change, and internal crosslinking (n_int_, shown in pink), which does not contribute. Based on this classification, we calculated the crosslinking efficiency (n_ext_/n_total_) to assess the patternability of the crosslinker. HDT, with a sulfur‐to‐sulfur distance of 0.95 nm, showed a low crosslinking efficiency of 0.167. In contrast, longer‐chain crosslinkers such as ODT (1.19 nm) and DDT (1.23 nm) exhibited higher crosslinking efficiencies of 0.357 and 0.64, respectively (Figure 2I; Figure S7, Supporting Information).

To determine the minimum crosslinker concentration required for achieving high retention ratios, fluorescence optical microscopy (F‐OM) analysis was conducted on PeNC patterns fabricated with HDT, ODT, and DDT as crosslinkers at various concentrations. (Figure 2J) The UV dose was fixed at 400 mJ cm^−2^ (with a 365 nm light source). As indicated by the retention ratio measurements, HDT did not form a clear pattern at 24.8 mM due to insufficient crosslinking. A concentration of at least 200 mM was required for HDT to produce clear patterns with high retention ratios. In contrast, long‐chain crosslinkers, ODT and DDT, required significantly lower concentrations to achieve pattern formation (24.8 mM for ODT and 5 mM for DDT) (Figure 2K). This trend aligns with previous studies showing that longer‐chain crosslinkers promote more effective interparticle crosslinking and solubility contrast.^[^ ^54^ ^]^ Based on these findings, ODT and DDT, which enabled effective pattern formation at minimal additive concentrations, were selected for the direct photocatalytic patterning process.

The patterning mechanism of the dithiol crosslinker, involving crosslinking through a thiol‐ene reaction, was confirmed through X‐ray photoelectron spectroscopy (XPS) and Fourier transform infrared (FTIR) spectral analyses. No peaks were observed in the XPS S 2p spectra of the pristine PeNC film, whereas a distinct peak was detected in the spectra of the PeNC–DDT film due to the presence of the dithiol crosslinker. DDT from the crosslinking remained even after UV irradiation and development, as confirmed by the presence of the S 2p peak (Figure S8, Supporting Information). The FTIR analysis showed that when a PeNC–ODT film was exposed to UV doses in the range 0–90 mJ cm^−2^ under a 365 nm UV source, the alkene peak at 3004 cm^−1^ from OA and OLA, as well as the thiol group peak at 2550 cm^−1^ from ODT, decreased in intensity with increasing UV dose (Figure 2L,M). This indicates that the main patterning mechanism is based on the thiol‐ene reaction leading to interparticle crosslinking, which is consistent with our previous result.^[^ ^37^ ^]^ The alkene peak for the PeNC–DDT film showed a similar trend (Figure S9, Supporting Information).

However, even with ODT and DDT as crosslinkers, the crosslinked PeNC films still incorporate substantial insulating ligands, such as OA and OLA with long aliphatic chains, which limited the performance of the PeNC‐LED. To further improve the electrical characteristics of the crosslinked PeNC films, we introduced the FLE approach. Conventional FLE methods using PbBr_2_/OA/OLA solutions after the patterning process were found to improve the optical properties but not the electrical properties.^[^ ^25^, ^32^ ^]^ To overcome these limitations, we developed an FLE process tailored for crosslinked PeNC films, employing a synergetic ligand mixture comprising phenethylammonium bromide (PEABr), guanidinium bromide (GABr), and 4‐methoxy‐phenethylammonium bromide (4‐MeO‐PEABr). PEABr has been widely utilized as an effective ligand for surface passivation of PeNCs.^[^ ^57^ ^]^ GABr ligands, with a compact, all‐conjugated molecular structure and smaller size than PEABr, enhance charge injection and provide denser passivation.^[^ ^58^ ^]^ 4‐MeO‐PEABr ligands containing a methoxy group contribute to the passivation of defect sites that are inaccessible to ammonium groups.^[^ ^59^ ^]^ This tailored FLE strategy enables the replacement of long‐chain native ligands with short‐chain alternatives while effectively passivating surface defects. This approach leads to concurrent improvements in both optical and electrical performance of the crosslinked PeNC films, thereby overcoming the limitations of conventional FLE methods. Furthermore, in contrast to conventional FLE processes, which can induce surface defects due to the use of polar solvents, the proposed FLE employs a nonpolar solvent as the main solvent, minimizing the formation of surface traps during the process.

**Figure 3:**
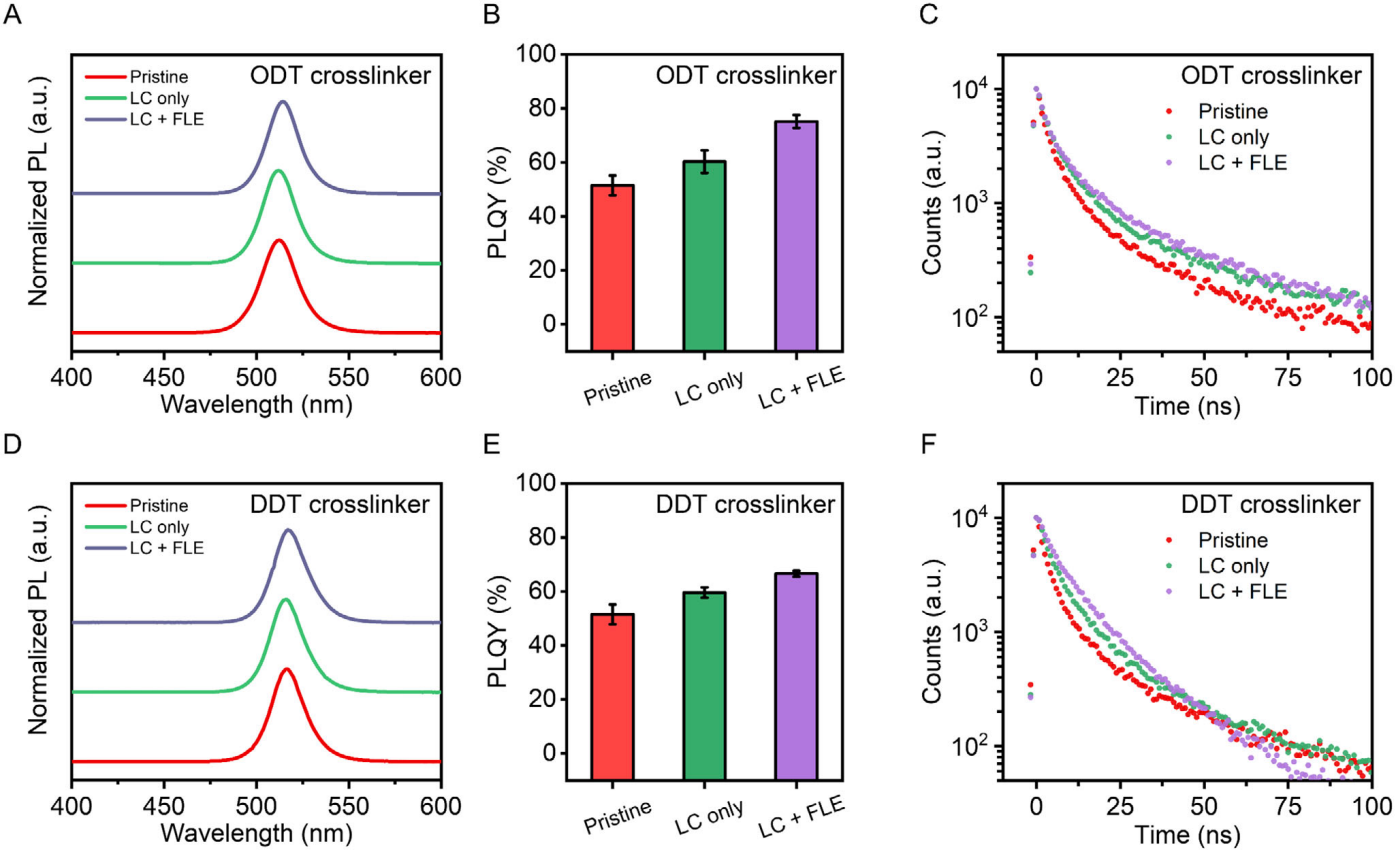
Optical characteristics of PeNCs with the LC and FLE processes: PL spectra, film PLQY, and PL decay curves of pristine PeNC films (pristine), LC‐only PeNC films, and LC + FLE‐treated PeNC films prepared using A‐C) ODT and D‐F) DDT as the thiol crosslinker. The error bars represent the standard deviations calculated from four independent runs.

We measured the PL emission spectra, PLQY, and time‐resolved PL to investigate the effects of the LC and FLE on the optical characteristics of PeNCs. After the LC process with ODT at a concentration of 24.8 mM and a UV dose of 200 mJ cm^−2^ (with a 365 nm light source), the emission peak of the PeNC remained unchanged, while a slight red shift of a few nanometers was observed following the FLE (**Figure** **3**A). This shift can be attributed to the supply of bromide sources and ligand exchange during FLE.

We investigated the PLQY of PeNC films at each step of the patterning process. The pristine PeNC film, which was synthesized without excess halide treatment and surface‐passivated with OA and OLA, exhibited a PLQY of 51.5%. After the LC process using ODT, the PLQY increased to 60.3% (Figure 3B), corresponding to a relative increase of >20% compared to the initial value. In contrast, in our previous study, the thiol crosslinker PTMP was found to be nondestructive toward PeNCs synthesized under excess bromide conditions, whereas its application to deficient pristine PeNCs resulted in a >20% decrease in PLQY.^[^ ^37^ ^]^

This difference can be attributed to variations in the surface ligand environment. Upon the introduction of thiol molecules, deprotonation of the thiol and subsequent protonation of surface‐bound oleate weaken the ligand–surface interaction, leading to oleate detachment.^[^ ^37^ ^]^ In PeNCs synthesized under excess bromide conditions, the surface is predominantly passivated with bromide ions and stabilized by oleylammonium ligands through electrostatic interaction, resulting in a low fraction of surface‐bound oleate and thereby reducing such oleate detachment.^[^ ^60^ ^]^ In contrast, bromide‐deficient pristine PeNCs, whose surfaces are primarily capped with oleate and OLA ligands, are more prone to oleate detachment. In such cases, PTMP shows limited surface accessibility due to its bulky structure and strong steric hindrance with other pre‐existing surface ligands, resulting in incomplete defect passivation and a reduction in PLQY. In comparison, ODT and DDT possess a linear alkyl chain that enables more favorable surface accessibility and facilitates efficient defect passivation, thereby leading to improved PLQY of the crosslinked PeNC films.

Then, we confirmed that the FLE process further enhanced the PLQY of crosslinked PeNC films. To assess its compatibility, we applied the FLE treatment process to both pristine and crosslinked PeNC films. The pristine and crosslinked PeNC films exhibited the PLQYs of 78.9% and 75.2% upon FLE, respectively, indicating that the FLE process is compatible for both films (Figure S10, Supporting Information). The increases in PLQY after LC and FLE processes are attributed to the reduced surface trap sites, which is supported by the increased average PL lifetime (Figure 3C; Table S1, Supporting Information). The same trend was observed when using DDT as the crosslinker (Figure 3D–F; Table S2, Supporting Information).

These results highlight that the combined LC and FLE processes enable precise and tunable control over the surface ligand environment of PeNCs (Figure S11, Supporting Information). The LC step introduces linear dithiol crosslinkers that form robust crosslinked networks while minimizing ligand loss. The subsequent FLE process replaces long‐chain ligands with short‐chain ammonium halides, resulting in denser surface passivation and further enhanced charge transport, as discussed in later sections.

**Figure 4:**
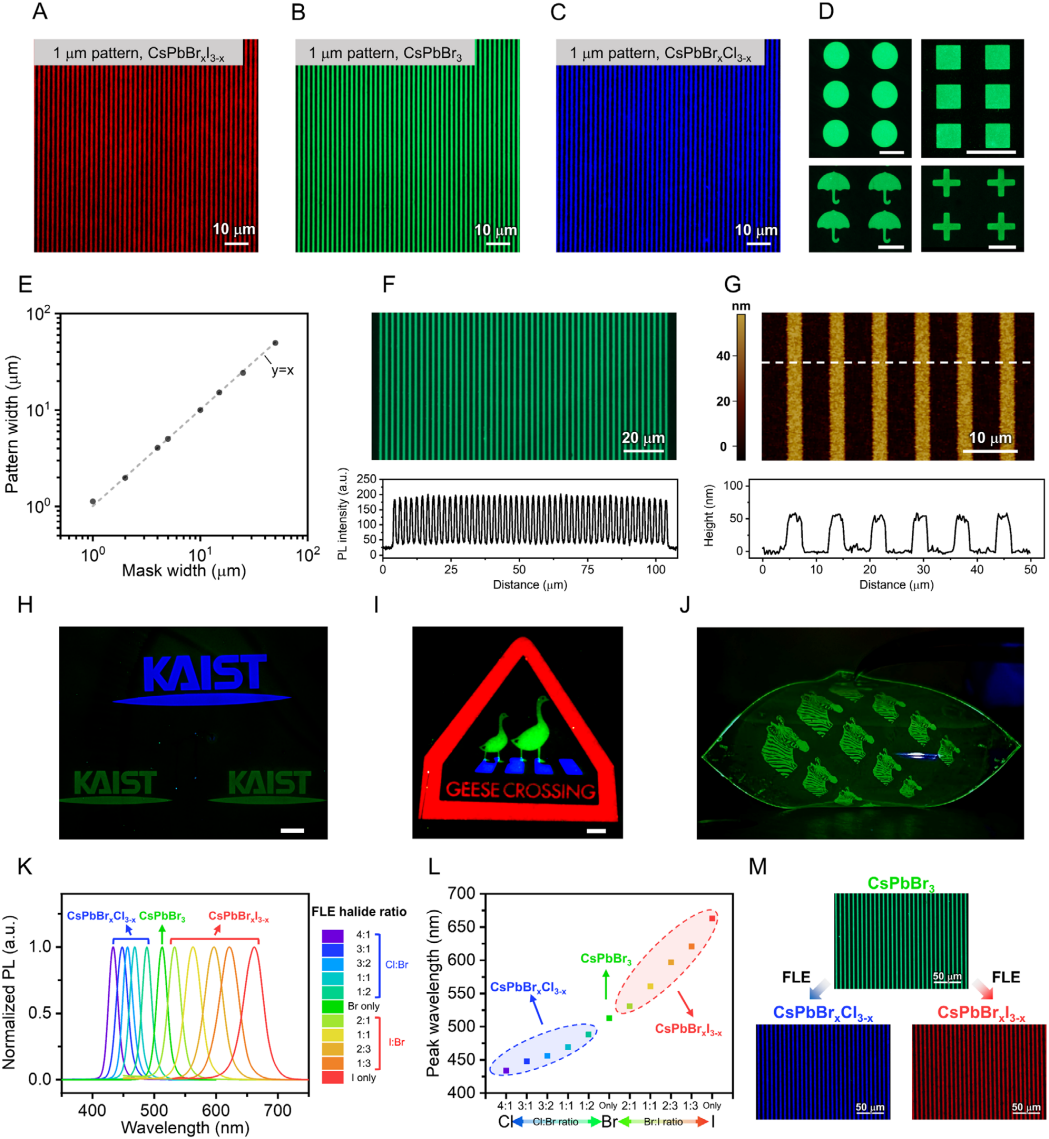
High‐resolution and multicolor patterns obtained via direct photocatalytic patterning: A‐C) F‐OM images of PeNC line patterns, A) CsPbBr_x_I_3−x_ PeNC pattern, B) CsPbBr_3_ PeNC pattern, and C) CsPbBr_x_Cl_3−x_ PeNC pattern. D) F‐OM images of PeNC patterns of various shapes (dots, squares, umbrellas, and crosses). Scale bar represents 100 µm. E) Accuracy analysis of direct photocatalytic patterning, along with a comparison of the photomask width with the fabricated PeNC pattern width. F) F‐OM image of a PeNC line pattern (top) and corresponding PL intensity profile (bottom) G) AFM image (top) and height profile along the white dashed line (bottom). H) GB PeNC pattern of “KAIST” letters achieved by repeated direct photocatalytic patterning, using CsPbBr_3_ PeNC for the green color and CsPbBr_x_Cl_3−x_ PeNC for the blue color. Scale bar represents 100 µm. I) RGB pattern of “geese crossing” achieved by repeated direct photocatalytic patterning using CsPbBr_3_ PeNC for the green color, CsPbBr_x_Cl_3−x_ PeNC for the blue color, and InP/ZnSe/ZnS QD for the red color. Scale bar represents 100 µm. J) Image of a PeNC pattern of zebras on a flexible substrate under UV exposure. All the samples were fabricated with ODT as the thiol crosslinker. K) PL spectra and L) corresponding peak wavelength of PeNC films after FLE with different halide compositions. M) F‐OM images of the high‐resolution red (CsPbBr_x_I_3−x_) PeNC, green (CsPbBr_3_), and blue (CsPbBr_x_Cl_3−x_) PeNC pattern, fabricated by the direct photocatalytic patterning process with ODT. The red and blue patterns were created through post‐patterning halide exchange using the FLE process.

We demonstrated high‐resolution patterning of red, green, and blue PeNCs via direct photocatalytic patterning using ODT and DDT as dithiol crosslinkers (**Figure** **4**). Specifically, ODT enabled the fabrication of CsPbBr_x_I_3−x_ (1 µm, red), CsPbBr_3_ (1 µm, green) and CsPbBr_x_Cl_3−x_ (1 µm, blue) PeNC line patterns, corresponding to resolutions of >10,000 PPI (assuming a single‐color pixel array) for green, red, and blue, surpassing the AR/VR display requirement (Figure 4A–C). PeNC array patterns with various shapes (dots, squares, umbrellas, and crosses) were also demonstrated (Figure 4D). Additionally, high‐resolution (≈2,500 PPI) square array pattern was achieved using DDT as a crosslinker (Figure S12, Supporting Information).

By assessing the patterning accuracy through the comparison of fabricated CsPbBr_3_ PeNC patterns (1–50 µm) with their corresponding photomask features, we confirmed that our method achieves high fidelity (Figure 4E; Figure S13, Supporting Information). Furthermore, we investigated the emission uniformity of the CsPbBr_3_ PeNC patterns. F‐OM imaging and line‐scan PL intensity profiling confirmed uniform emission characteristics in the 2‐µm and 4‐µm patterns (Figure 4F; Figure S13, Supporting Information). Atomic force microscopy (AFM) analysis showed that the average surface roughness *R_a_* of the pristine CsPbBr_3_ PeNC film was calculated to be 3.5 nm, whereas the crosslinked film exhibited a slightly reduced *R_a_* of 2.3 nm, suggesting minimal morphological alterations (Figure S14, Supporting Information). X‐ray diffraction (XRD) analysis further confirmed that the crystal structure of PeNCs was preserved after the patterning process (Figure S15, Supporting Information). Height profile measurement of fabricated high‐resolution CsPbBr_3_ PeNC line pattern verified the uniform feature size of 3 µm with a thickness of ≈50 nm (Figure 4G).

To demonstrate the orthogonality of the direct photocatalytic patterning process using dithiol crosslinkers, we fabricated a green‐blue (GB) pattern. Using ODT as the crosslinker, blue CsPbBr_x_Cl_3−x_ PeNCs were patterned into various resolutions and shapes (Figure S16, Supporting Information). Sequential direct photocatalytic patterning enabled the formation of GB patterns by integrating blue CsPbBr_x_Cl_3−x_ and green CsPbBr_3_ PeNCs. The clear distinction between the green and blue PeNC regions suggests that ODT effectively passivated the PeNC surface, suppressing undesired halide exchange during the patterning process (Figure 4H).

Furthermore, we demonstrated the versatility of dithiol‐based direct photocatalytic patterning across diverse nanomaterials beyond PeNCs. Using ODT as a crosslinker, we successfully fabricated a whale pattern of InP‐based core/shell QDs (Figure S17, Supporting Information). This approach also enabled the fabrication of red‐green‐blue (RGB) patterns through a consecutive patterning process involving red InP‐based QD and green/blue PeNC patterns (Figure 4I; Figure S18, Supporting Information).

Moreover, direct photocatalytic patterning using dithiols was applicable not only to rigid substrates such as glass and Si but also to flexible substrates. Figure 4J presents a zebra pattern fabricated on a thermoplastic polyurethane (TPU) substrate, highlighting the adaptability of this method for flexible optoelectronics.

Additionally, the FLE process enables post‐patterning halide exchange, offering precise control over the emission spectrum of PeNC features. In the previously fabricated GB pattern using the sequential process, halide exchange was effectively suppressed due to surface passivation by ODT, which preserved the initial halide composition. In contrast, the FLE process is specifically designed to permit compositional tuning: an initial acetate treatment detaches native ligands and exposes the nanocrystal surface, facilitating subsequent halide exchange. Treatment with a high concentration of ammonium halides, chosen for their strong binding affinity, then enables halide incorporation while simultaneously re‐establishing surface passivation. To further investigate the compositional tunability enabled by the FLE process, we treated green CsPbBr_3_ PeNC films with ligand solutions containing various halide compositions (Figure 4K,L). As a result, the emission wavelength was tunable across a wide spectral range, from 434 nm (violet) to 663 nm (red), covering the majority of the visible spectrum. This spectral tunability was also applicable to patterned PeNCs: 4‐µm‐wide green CsPbBr_3_ line patterns were successfully converted into red CsPbBr_x_I_3−x_ or blue CsPbBr_x_Cl_3−x_ patterns via FLE process, without compromising structural uniformity (Figure 4M).

**Figure 5:**
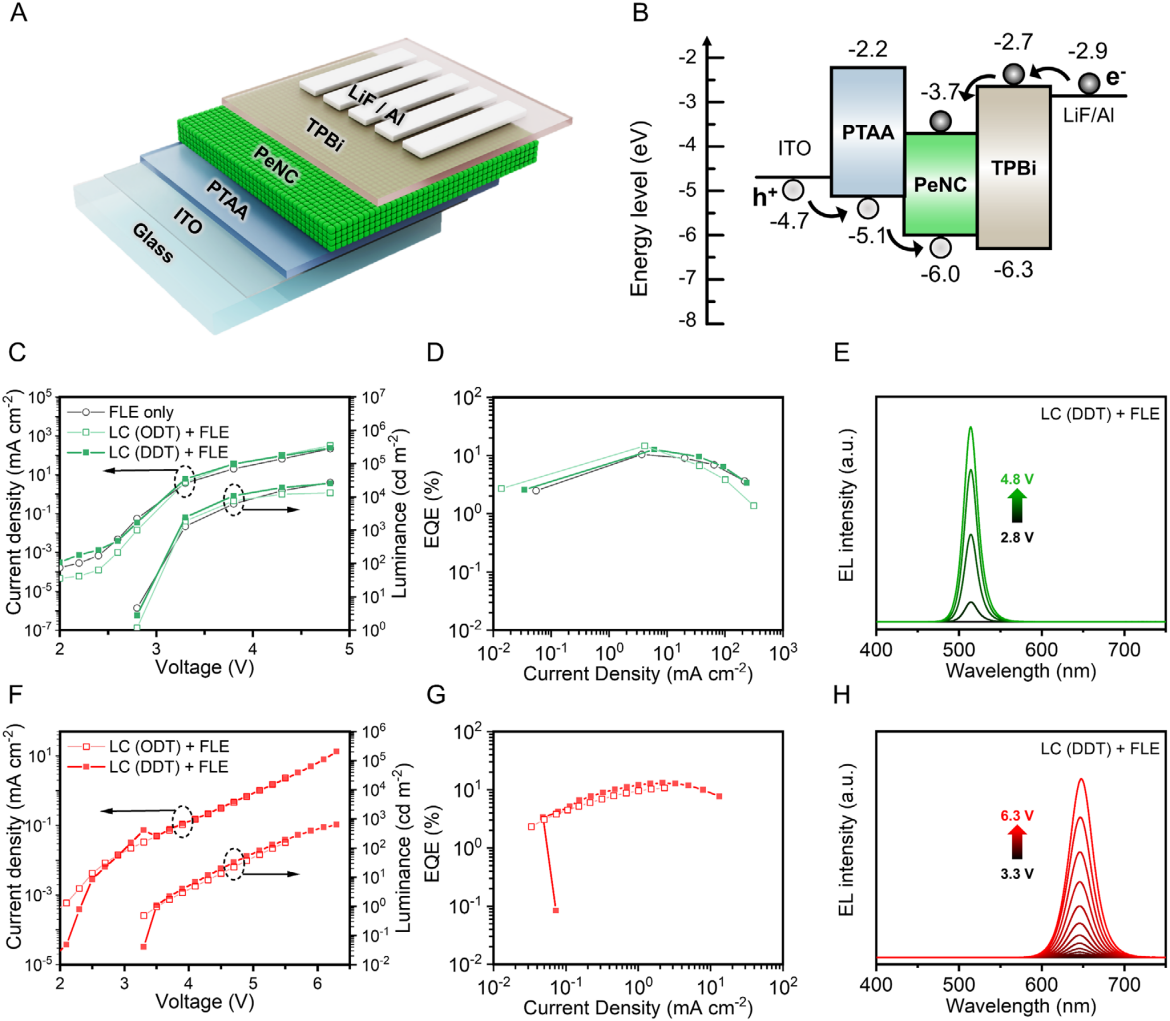
Electrical characteristics of PeNC‐LEDs prepared using a dual strategy: A) Schematic of the PeNC‐LED structure. B) Energy band structure of the PeNC‐LED. C) Current density–voltage and luminance–voltage characteristics and D) EQE–current density characteristics of green CsPbBr_3_ PeNC LEDs, including FLE‐applied PeNC film (FLE only), FLE‐applied crosslinked PeNC film with ODT crosslinker (LC (ODT) + FLE), and FLE‐applied crosslinked PeNC film with DDT crosslinker (LC (DDT) + FLE). E) EL spectra of the FLE‐applied crosslinked PeNC film with DDT crosslinker (LC (DDT) + FLE) under different voltage levels. F) Current density–voltage and luminance–voltage characteristics and G) EQE–current density characteristics of FLE‐applied crosslinked red CsPbBr_x_I_3−x_ PeNC LEDs prepared using ODT and DDT as the thiol crosslinkers. H) EL spectra of the FLE‐applied crosslinked PeNC film with the DDT crosslinker (LC (DDT) + FLE) under different voltage levels.

Finally, to evaluate the impact of our dual strategy (ODT/DDT‐based LC and FLE) on EL characteristics, we fabricated PeNC‐LEDs by applying FLE to a crosslinked green CsPbBr_3_ PeNC emission layer (**Figure** **5**). The PeNC‐LED device structure consisted of glass/indium tin oxide (ITO)/PTAA (5 nm)/PeNCs (50 nm)/2,2′, 2″‐(1,3,5‐Benzinetriyl)‐tris(1‐phenyl‐1‐H‐benzimidazole) (TPBi) (40 nm)/lithium fluoride (LiF) (1 nm)/Al (75 nm) (Figure 5A,B). For systematic investigation, four different CsPbBr_3_ PeNC‐LEDs were compared: 1) pristine PeNC, 2) FLE only, 3) LC (ODT) + FLE, and 4) LC (DDT) + FLE. The pristine CsPbBr_3_ PeNC‐LED device exhibited an *EQE_max_* of 1.49% and a *L_max_* of 686 cd m^−2^, comparable to those in previous studies^[^ ^25^, ^37^ ^]^ and ascribed to the presence of insulating long‐chain ligands which hinder charge injection and transport (Figure S19, Supporting Information). However, the application of FLE along significantly improved EL characteristics, increasing the *EQE_max_* to 10.4%, and the *L_max_* to ≈27,000 cd m^−2^, representing 7‐fold and 39‐fold enhancements, respectively. These improvements are attributed to the synergistic effects of incorporating a series of short‐chain ammonium halide ligands including phenethylammonium halide (PEAX), guanidinium halide (GAX), and 4‐methoxy‐phenethylammonium halide (4‐MeO‐PEAX) in the ligand exchange process. The exchanged ligands effectively passivated the PeNC surface, contributing to the improved EQE observed in FLE‐treated PeNC‐LEDs. Also, short‐chain ammonium halide ligands further facilitated charge transport, resulting in higher current density relative to pristine PeNC‐LEDs and leading to improved luminance. Devices crosslinked with ODT or DDT exhibited similar current density–voltage characteristics to the FLE‐only device (Figure 5C), indicating that crosslinking did not introduce charge transport barriers. Furthermore, the turn‐on voltages of the crosslinked PeNC‐LEDs (2.7–2.8 V) were comparable to that of the FLE‐only device (2.7 V), confirming that the crosslinked emission layers preserved efficient charge injection and transport properties (Table S3, Supporting Information). Notably, the crosslinked devices exhibited *EQE_max_* of 14.7% and *L_max_* of ≈25,400 cd m^−2^ (Table S3, Supporting Information), achieving the highest *EQE_max_* and *L_max_* reported among green CsPbBr_3_ PeNC‐LEDs following direct optical patterning.^[^ ^25^, ^37^, ^42^, ^43^ ^]^ Interestingly, PeNC‐LEDs with both LC and FLE exhibited higher EQE values than that of the FLE‐only device. The improved EL characteristics can be attributed to higher PLQY values of crosslinked and FLE‐treated PeNCs (Figure 3B,E). These results confirm that ODT and DDT act not only as optimal crosslinkers but also as surface ligands improving defect passivation on the PeNC surfaces, preserving both the electrical and optical properties of PeNCs in synergy with FLE strategy. Across multiple fabricated devices, a consistently high EQE was observed in FLE‐treated crosslinked PeNC‐LEDs (Figure S20, Supporting Information). During device operation, the EL spectra of green FLE‐treated crosslinked PeNC‐LEDs exhibited a stable emission peak without significant spectral shift or broadening (Figure 5E; Figure S21, Supporting Information).

We further investigated the spectral tunability of our dual‐strategy direct photocatalytic patterning by fabricating mixed‐halide red CsPbBr_x_I_3−x_ PeNC‐LEDs via FLE‐induced halide exchange. Following the fabrication of the green crosslinked PeNCs, a ligand solution containing iodine ions (as described in the Experimental Section) was employed in the FLE process, enabling the conversion into mixed‐halide red PeNCs. The LC (ODT) + FLE red CsPbBr_x_I_3−x_ PeNC‐LED exhibited *EQE_max_* of 10.8% and *L_max_* of 155 cd m^−2^, and the corresponding DDT variant achieved *EQE_max_* of 13.1% and *L_max_* of 637 cd m^−2^; these results represent the first demonstration of red PeNC‐LEDs fabricated with PeNC inks compatible with direct optical patterning processes (Figure 5F,G). The EL spectra remained stable at high operating voltages, without any notable shift or broadening (Figure 5H; Figure S22, Supporting Information). Spectral instability in mixed‐halide perovskites typically arises from halide ion migration through defect‐mediated pathways.^[^ ^61^, ^62^ ^]^ Therefore, the observed spectral stability further confirms that the PeNC surfaces were densely passivated by the dual strategy, mitigating defect‐mediated halide migration.

These results demonstrate that our dual‐strategy direct photocatalytic patterning enables high‐resolution patterning of RGB PeNC films while preserving their optical and electrical properties, ensuring compatibility with high‐efficiency PeNC‐LED fabrication processes. To the best of our knowledge, the crosslinked CsPbBr_3_ PeNC‐LEDs exhibit one of the highest reported *EQE_max_* and *L_max_* among CsPbBr_3_ PeNC‐LEDs fabricated using photopatternable PeNC inks (Table S4, Supporting Information). While our study suggests promising routes for achieving high‐resolution, high‐efficiency PeNC‐LEDs, the realization of high‐efficiency PeNC‐LED arrays with micrometer‐scale pixel sizes remains an open challenge. Furthermore, the integration of such high‐resolution PeNC‐LEDs with an active‐matrix backplane has yet to be demonstrated. Therefore, future research should focus on optimizing the device architecture and addressing integration challenges to enable the realization of perovskite‐based immersive AR/VR devices.

## 3 Conclusion

In conclusion, we developed a dual strategy that enables both high‐resolution, nondestructive direct photocatalytic patterning and the fabrication of efficient crosslinked PeNC‐LEDs. The first strategy is to advance a direct photocatalytic patterning method suitable for LED fabrication. We systematically optimized the design of thiol crosslinkers and identified ODT and DDT as optimal candidates due to their solvent compatibility, colloidal stability, and patternability, which enabled an effective LC process. This approach enabled the fabrication of high‐resolution, uniform PeNC patterns with high fidelity while preserving their optical properties, and successfully demonstrated RGB orthogonal patterns. The second strategy employed FLE, which improved the optical and electrical properties of the PeNCs by controlling surface ligands and enabled color conversion through halide composition tuning. An efficient green crosslinked CsPbBr_3_ PeNC‐LED, prepared using the dual strategy, exhibited an *EQE_max_* of 14.7% and a *L_max_* of ≈25,400 cd m^−2^. Furthermore, the FLE approach facilitated the first ever demonstration of a red crosslinked PeNC‐LED with an *EQE_max_* of 13.1%. Our dual strategy provides an effective approach for realizing high‐resolution, high‐performance pixelated devices for next‐generation AR/VR displays.

## 4 Experimental Section

### Ligand Crosslinking (LC) Process

Thiol crosslinkers (ODT or DDT) were dissolved in octane. A formulated ink was prepared by mixing the desired amount of the thiol crosslinker solution with the PeNC solution, which had a concentration of ≈40 mg mL^−1^ in octane. This ink was then spin‐coated onto a substrate (2,000 rpm, 60 s). The PeNC–thiol crosslinker film was aligned with a pre‐made photomask and exposed to UV light (365 nm) at the desired dose. The UV‐unexposed region was developed using octane through a spin‐coating process.

### Fabrication of PeNC‐LED using Film‐State Ligand Exchange (FLE)

An acetate solution was prepared by dissolving 0.08 g of lead nitrate (Pb(NO_3_)_2_) and acetic acid in 10 mL of methyl acetate and sonicating for 20 min. After the solution reached saturation, it was centrifuged at 4000 rpm for 5 min to remove excess Pb(NO_3_)_2_. For the ligand solution, we dissolved 0.033 g of PEABr, 0.023 g of GABr, 0.038 g of 4MeO‐PEABr, 0.026 g of PEACl, 0.016 g of GACl, 0.031 g of 4MeO‐PEACl, 0.041 g of PEAI, 0.033 g of GAI, and 0.046 g of 4MeO‐PEAI in 5 mL of butanol (BuOH) respectively. Subsequently, the precursors were dissolved in octane as follows: 1 mL of the PEABr precursor, 0.5 mL of the GABr precursor, and 0.5 mL of the 4MeO‐PEABr precursor were dissolved in 3 mL of octane for the green PeNCs; 1 mL of PEAI, 0.5 mL of GAI, and 0.5 mL of 4MeO‐PEAI precursors were dissolved in 3 mL of octane for the red PeNCs; 0.75 mL of PEACl, 0.25 of PEABr, 0.375 mL of GACl, and 0.125 mL of GABr, 0.375 mL of 4MeO‐PEACl, and 0.125 mL of 4MeO‐PEABr precursors dissolved in 3 mL of octane for the blue PeNCs.

A solution of PTAA (4 mg mL^−1^ in chlorobenzene) was spin‐coated onto ITO‐patterned glass substrates at 5,000 rpm for 30 s; the resulting film was then baked in a glove box at 100 °C for 5 min. For FLE‐only PeNC‐LEDs, the purified PeNC solution was spin‐coated at 2,000 rpm for 60 s; the resulting film was then annealed in a glove box at 80 °C for 5 min. For LC + FLE‐treated PeNC‐LEDs, the formulated ink was spin‐coated at 2,000 rpm for 60 s, exposed to UV light, spin‐coated with the mother solvent, and then annealed under the same conditions as the FLE‐only device (formulated ink concentrations: ODT, 49 mM; DDT, 18.6 mM; UV dose for both: 120 mJ cm^−2^). The acetate solution was applied to the PeNC films for 5 s and dried by spinning at 2,500 rpm for 30 s. Subsequently, ligand solutions were applied for 10 s for green and red LEDs; they were then dried through spinning at 2,500 rpm for 30 s. The ligand‐exchanged PeNC films were rinsed by briefly soaking in octane for 5 s, then spun for 30 s for complete drying. Finally, a 40 nm TPBi layer was deposited as the electron transport layer, followed by a LiF/Al layer (1/75 nm) as the top electrode, using thermal evaporation under high‐vacuum conditions (2 × 10^−7^ Torr).

## Conflict of Interest

S.M., S.J.P., and H.C. are inventors on patent applications KR10‐2023‐0051499 and KR10‐2023‐0076415, which are partially based on this work. The authors declare that they have no other conflict of interest.

## Supporting information



Supporting Information

## Data Availability

The data that support the findings of this study are available from the corresponding author upon reasonable request.
