# Supplementary material for: Dual‐Strategy Direct Photocatalytic Patterning for Efficient Perovskite Nanocrystal LED Displays
Source: Adv Mater. 2025 Jul 31;37(42):e08217. doi: 10.1002/adma.202508217 (PMC12548516; doi:10.1002/adma.202508217)
Supplement: Supplementary file 1 — Supporting Information [file ADMA-37-e08217-s001.pdf]

ADVANCED MATERIALS

Supporting Information

for *Adv. Mater.*, DOI 10.1002/adma.202508217

Dual-Strategy Direct Photocatalytic Patterning for Efficient Perovskite Nanocrystal LED Displays

Seongkyu Maeng, Junho Kim, Taehyun Kim, Seyun Lee, Seunghee Han, Sun Jae Park, Changjo Kim, Jihan Kim, Jung-Yong Lee and Himchan Cho**

Supporting Information

Title: Dual-Strategy Direct Photocatalytic Patterning for Efficient Perovskite Nanocrystal LEDs

Seongkyu Maeng, Junho Kim, Taehyun Kim, Seyun Lee, Seunghee Han, Sun Jae Park, Changjo Kim, Jihan Kim, Jung-Yong Lee, Himchan Cho**

Figs. S1 to S22

Tables S1 to S4

Experimental Method

1. Chemicals

The following chemicals, purchased from Sigma-Aldrich Co., Ltd, were used in this study: 1,6-Hexanedithiol (HDT, >97%, TCI), 1,8-octanedithiol (ODT, >97%, TCI), 1,10-decanedithiol (DDT, >98%, TCI), 3,6-dioxa1,8-octanedithiol (DODT, >97%, TCI), 1-octanethiol (OT, >98.5%, Aldrich), pentaerythritol tetrakis(3-mercaptopropionate) (PTMP, >95%, Aldrich), trimethylolpropane tris (3-mercaptopropionate) (TTMP, >95%, Aldrich), lead (II) bromide ($PbBr_2$, trace metals basis, 99.999%), cesium carbonate (Cs_2CO_3, trace metals basis, 99.995%), oleic acid (OA, technical grade, 90%), oleylamine (OLA, technical grade, 70%), 1-octadecene (ODE, technical grade, 90%), acetic acid (≥99.7%), methyl acetate (MeOAc, anhydrous, 95%), ethyl acetate (EtOAc, anhydrous, 99.8%), 1-butanol (BuOH, anhydrous, 99.8%), hexane (laboratory reagent, 95%), octane (anhydrous, 99%), phenethylammonium iodide (PEAI, ≥98%), phenethylammonoum bromide (PEABr, ≥98%), phenethylammonium chloride (PEACl, ≥98%), guanidinium iodide (GAI, ≥99%), guanidinium bromide (GABr, ≥98%), guanidinium chloride (GACl, ≥98%), lead (II) nitrate ($Pb(NO_3)_2$, trace metals basis, 99.999%), poly[bis(4-phenyl)(2,4,6-trimethylphenyl)amine (PTAA), lithium fluoride (LiF, trace metals basis, 99.99%). Additionally, 4-methoxy-phenylammonium iodide (4MeO-PEAI, >99%), 4-methoxy-phenylammonium bromide (4MeO-PEABr, >99%), and 4-methoxy-phenylammonium chloride (4MeO-PEACl, >99%) were purchased from Greatcell Solar Materials Pty., Ltd.

2. Synthesis of PeNCs

A Cs–oleate precursor was prepared by combining 0.5 g of Cs_2CO_3, 2 mL of OA, and 50 mL of ODE in a 100 mL three-neck flask. The mixture was degassed at a temperature of 120 °C for 1 h to ensure complete reaction between Cs_2CO_3 and OA, as indicated by the solution becoming transparent. After degassing, the flask was purged with Ar gas and maintained at a temperature of 100 °C.

Subsequently, $PbBr_2$ (0.69 g) in 50 mL of ODE was prepared in a 100 mL three-neck flask. The mixture was degassed at a temperature of 120 °C for 1 h. Moreover, 5 mL of OA and 5 mL of OLA were pre-heated to 70 °C. After degassing, the flask was purged with Ar gas, and the pre-heated OA and OLA were added. The mixture was further degassed at a temperature of 120 °C until the solution was clear. The flask was purged with Ar gas, and the temperature was increased to 165 °C. In this step, 8 mL of the prepared Cs–oleate precursor was rapidly injected into the flask, and the reaction was allowed proceeded for 5 s. After reaction, the flask was quenched in an ice bath.

The PeNC solution was purified by mixing the crude solution with MeOAc in a volume ratio of 1:2 inside a glove box. The mixture was centrifuged at 8000 rpm for 10 min, and the resulting precipitate was redispersed in hexane. The same purification process was repeated twice. In the final purification step, the precipitate was redispersed in 6 mL of octane.

3. Molecular dynamics (MD) simulations

To investigate the effect of crosslinker chain length on crosslink formation between PeNC surfaces, we performed classical molecular dynamics (MD) simulations. The simulation system included only surface ligands and crosslinkers, omitting the PeNC for simplicity. To mimic the bounded ligands on PeNC surfaces, the terminal oxygen atom of each oleic acid was fixed during the simulation, while the rest of the molecule was fully flexible.

The simulation box was set to 30 Å × 30 Å × 30 Å, with periodic boundary conditions in the x and y directions, and non-periodic boundaries in the z direction to reflect the physical boundaries of the surfaces. The typical surface ligand composition of PeNCs includes both oleic acid and oleylamine, but for computational convenience, only oleic acid was used. While the reported surface ligand density of PeNCs is ~1.7 nm^{-2} [1], we assumed a density of 1.0 nm^{-2} due to ligand loss during the harsh purification conditions and film fabrication process. Based on this density, 9 oleic acid molecules were placed on each of the two opposing sides (a total of 18 molecules) at a distance of 2.5 nm, as based on previous work.[2] Six crosslinker molecules (corresponding to a 3:2 crosslinker-to-ligand ratio) were randomly placed in the space using Packmol.[3] All molecular structures, including oleic acid and crosslinkers, were obtained from PubChem.[4] The double bond of oleic acid was replaced by a C–C bond for simplicity, and the nearest hydrogen atom was designated as the bond-forming site. A bond was considered to form when a sulfur atom from the crosslinker approached within 5 Å of the carbon atom bonded to the designated hydrogen atom on a surface ligand.

All MD simulations were performed using LAMMPS.[5] The General Amber Force Field (GAFF) was used throughout, as it is suitable for a wide range of organic molecules.[6] The bond formation mechanism was implemented using a distance-based algorithm which dynamically applies appropriate force field parameters to the bonds formed during the simulation.[7,8] Precomputed GAFF parameters for bonded oleic acid-crosslinker structures were stored in a lookup table and assigned in real time when bond formation occurred.

The simulation proceeded as follows. First, a 100 ps NVE simulation was conducted for initial relaxation, followed by 100 ps of NVT equilibration at 298 K. Bond formation was subsequently evaluated by checking whether the distance between a sulfur atom from a crosslinker and the carbon atom bonded to the designated hydrogen on a surface ligand fell

below 5 Å. Next, the system was re-equilibrated using 10 ps of NVE and 10 ps of NVT at 298 K. This process was repeated until no new bond formation occurred for 10 consecutive cycles. Due to the irreversible nature of bonding, initial crosslinker positions could influence the outcomes. Therefore, five independent simulations were performed, and the three cases with the highest number of reacted surface sites were used to calculate the average crosslinking efficiency, excluding the two least-reactive cases to minimize bias.

4. Characterization Techniques

Transmission electron microscopy (TEM) was performed using a Tecnai G2 F30 S-Twin (FEI company). The TEM sample was prepared by drop-casting the PeNC solution onto an FCF200-Cu TEM grid. Absorption spectra were measured using a UV–Vis spectrophotometer (JASCO V-770). Fluorescence optical microscopy (F-OM) measurements were conducted using a Nikon Eclipse 80i with X-cite Xylis LED excitation. X-ray photoelectron spectroscopy (XPS) spectra were measured in a nitrogen atmosphere using a Sigma Probe (Thermo VG Scientific) with an Al source (1486.7 eV). Fourier transform infrared (FTIR) measurements of the film were conducted on a Nicolet iS50 (Thermo Fisher Scientific), with a range of 4000–400 cm^{-1} and resolution of 4 cm^{-1}, using attenuated total reflection (ATR). The photoluminescence quantum yield (PLQY) and photoluminescence (PL) emission spectra of the PeNC films were measured using a spectrofluorometer (JASCO FP-8550) with an integrating sphere. The film PLQY was measured by first obtaining the baseline with only the substrate; subsequently, the PL emission spectra of the PeNC film were measured. From these two sets of data, the spectrofluorometer peak integration value of the substrate (I_{origin}), the peak integration of the sample (I_{sample}), and the PL emission peak integration value of the sample ($I_{emission}$) were calculated. The PLQY was calculated using the formula: $I_{emission}$ / (I_{origin} - I_{sample}) × 100%. The excitation wavelength for the PL measurement was 360 nm, and the emission wavelength range was 300–600 nm. Time-resolved photoluminescence (TrPL) measurements of the PeNC films were performed with a Fluorolog-QM (HORIBA). Atomic force microscopy (AFM) measurements were conducted using an NX10 (Park Systems). X-ray diffraction (XRD) measurements of the PeNC films were performed with SmartLab (RIGAKU).

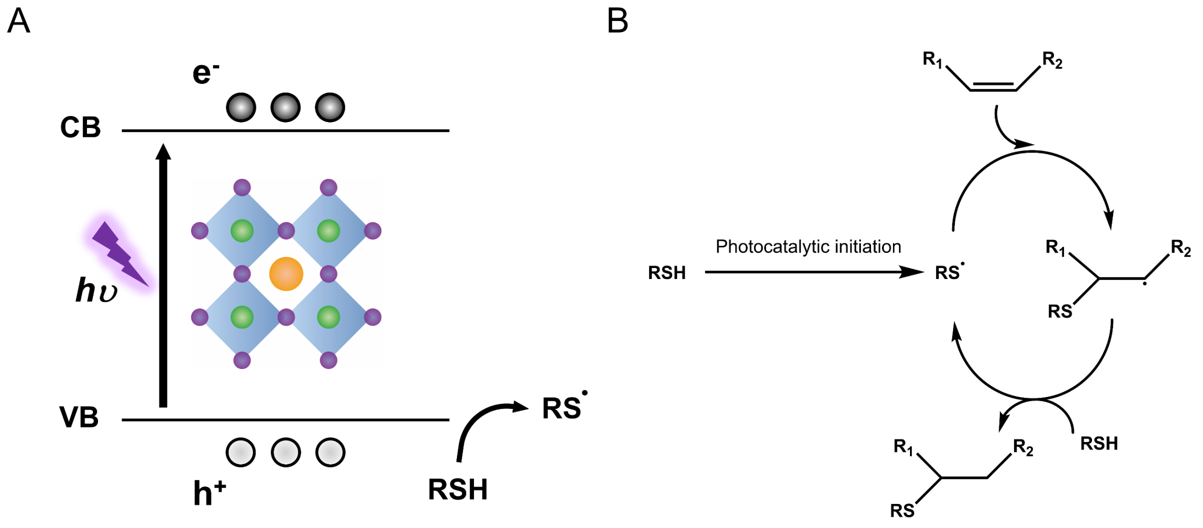

Figure S1. Schematic diagram describing direct photocatalytic patterning process. **(A)** photoexcitation of charge carriers in PeNCs, leading to the generation of thiyl radicals. **(B)** Proposed thiol–ene reaction mechanism during the direct photocatalytic patterning.[9] CB: conduction band; VB: valence band.

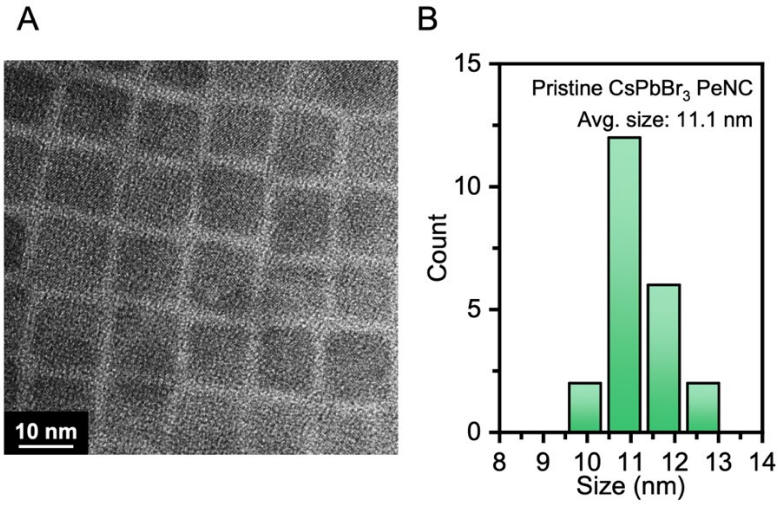

Figure S2. (A) Transmission electron microscopy (TEM) image and **(B)** size distribution of green $CsPbBr_3$ PeNCs. The average size of $CsPbBr_3$ PeNCs is 11.1 nm.

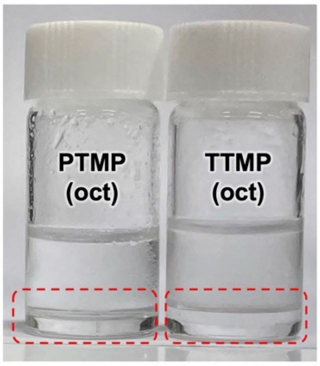

Figure S3. Images of PTMP and TTMP dispersed in octane. The red dashed box indicates phase separation between the solvent and thiol molecules.

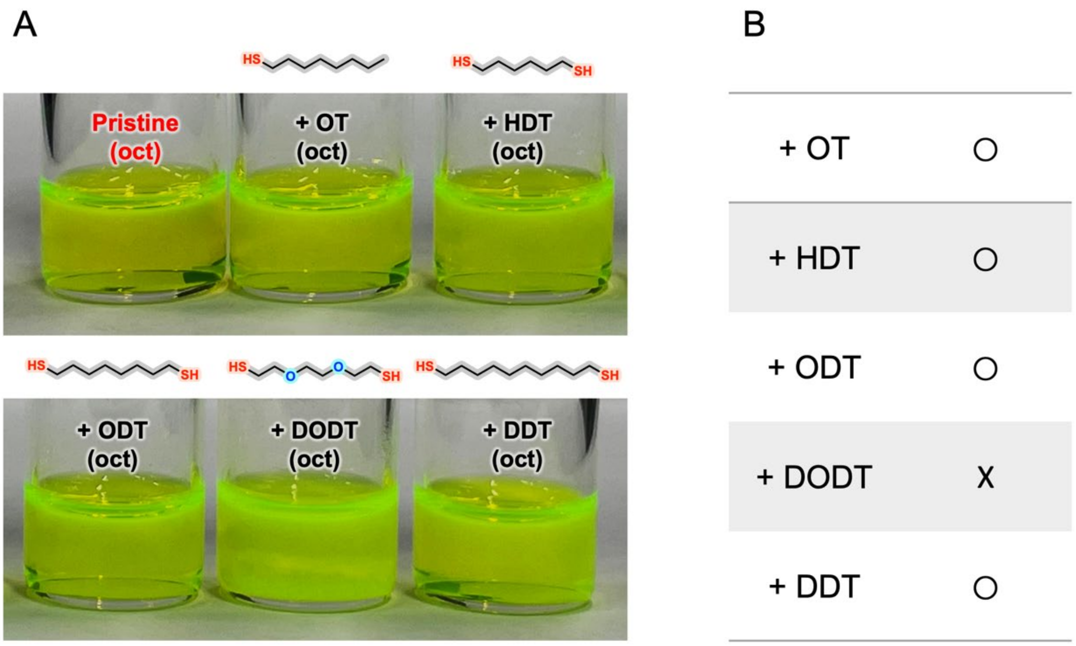

Figure S4. (A) Images of pristine green $CsPbBr_3$ PeNC solution and formulated inks prepared by mixing green $CsPbBr_3$ PeNC solution with various thiol molecules (OT, HDT, ODT, DODT, and DDT); all of them were dispersed in octane solvent. **(B)** Summary of the colloidal stability of the formulated inks, where “O” and “X” indicate high and low colloidal stabilities.

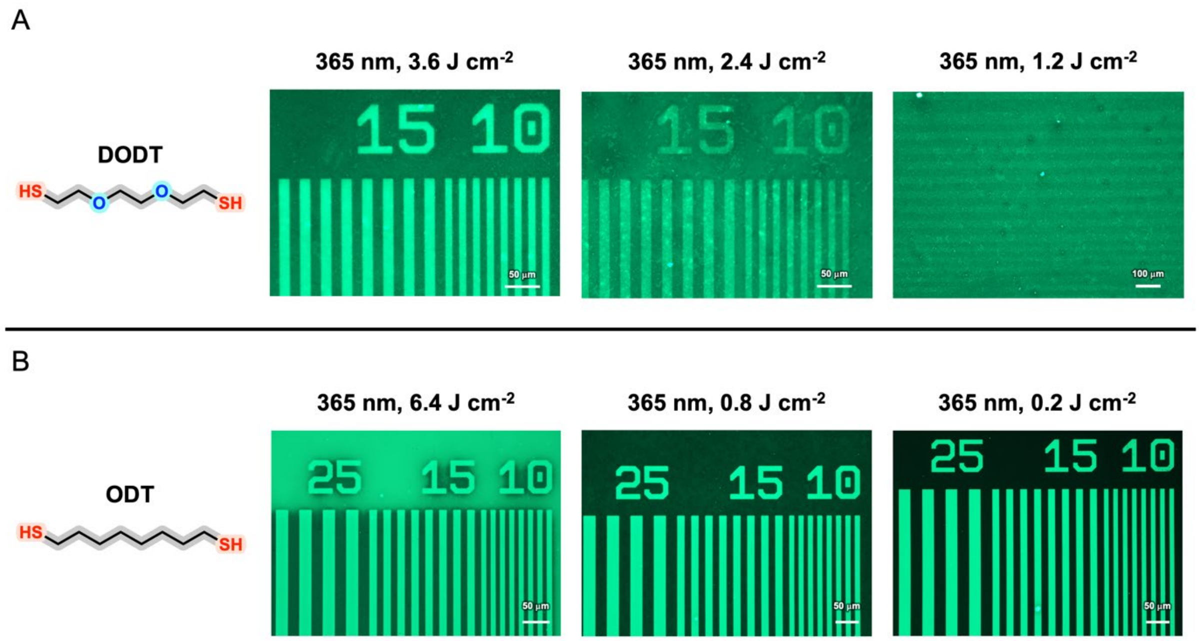

Figure S5. Fluorescence-optical microscopy (F-OM) images of green $CsPbBr_3$ PeNC patterns created via the direct photocatalytic patterning process using **(A)** DODT and **(B)** ODT as the crosslinker under different UV dose conditions.

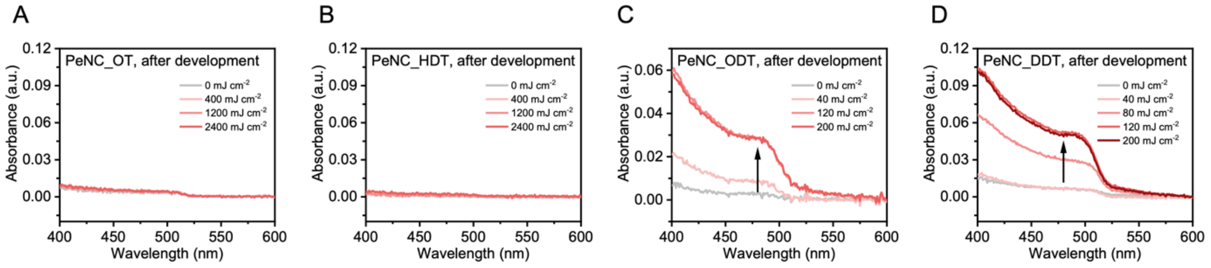

Figure S6. (A-D) Absorption spectra of PeNC–OT, PeNC–HDT, PeNC–ODT, and PeNC–DDT films after the development process under varying UV dose conditions.

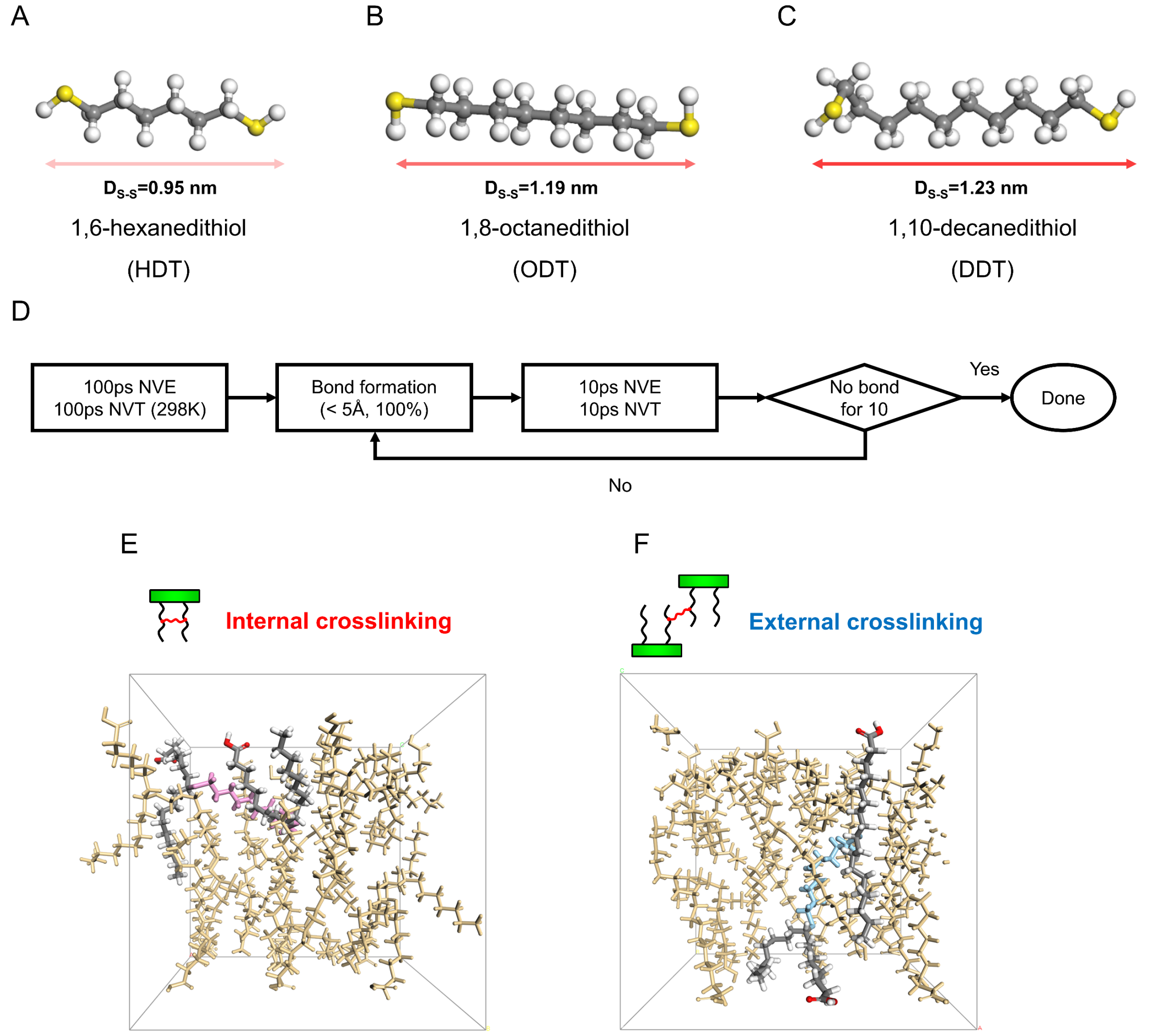

Figure S7. (A-C) Molecular structures of HDT, ODT, and DDT. **(D)** Flowchart of crosslinked system formation. Representative conformations of **(E)** internal crosslinking and **(F)** external crosslinking in MD simulations. Crosslinkers involved in internal crosslinking are shown in pink, while those involved in external crosslinking are shown in sky blue. Since the actual PeNC surface is larger, the x and y directions are assumed to be periodic. Ligands appearing outside the simulation space are shown to illustrate molecular connectivity and improve visual clarity.

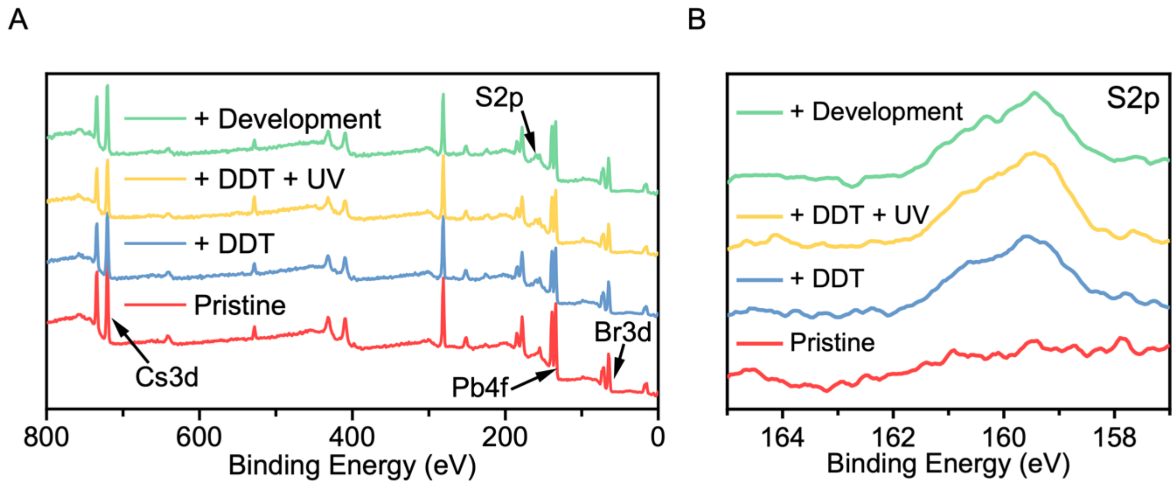

Figure S8. (A-B) X-ray photoelectron spectroscopy (XPS) spectra of a pristine green $CsPbBr_3$ PeNC film and a PeNC–DDT film at each patterning step. The UV dose is 400 mJ cm^{-2}.

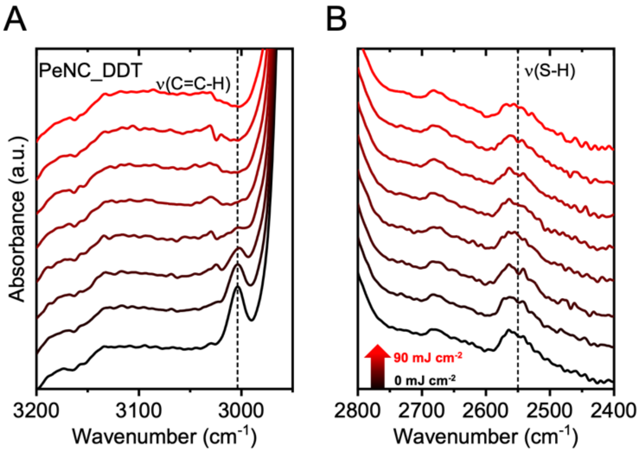

Figure S9. (A-B) FTIR spectra of the PeNC–DDT film under different UV exposure doses (0 to 90 mJ cm^{-2}).

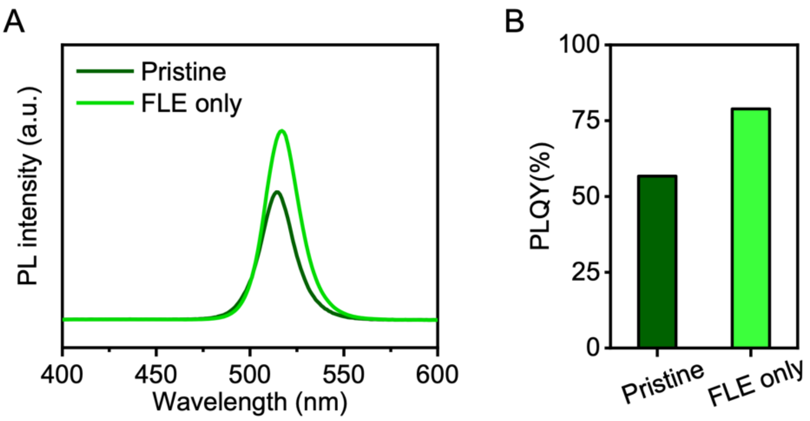

Figure S10. (A) PL spectra and **(B)** film PLQY of pristine PeNC film (Pristine), and FLE-applied PeNC film (FLE only).

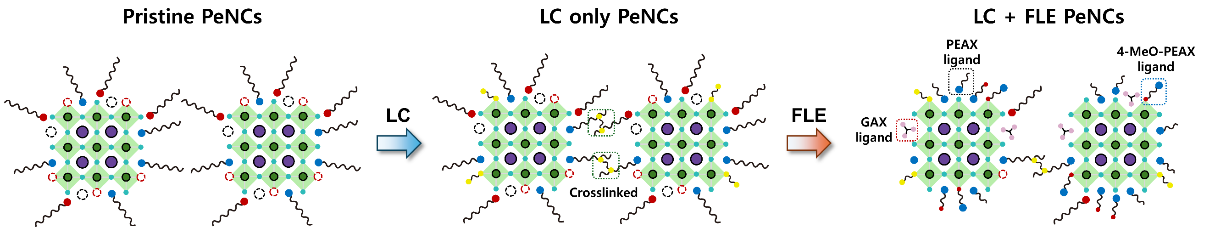

Figure S11. Schematic illustration of surface ligand modification of PeNCs through the LC and FLE processes.

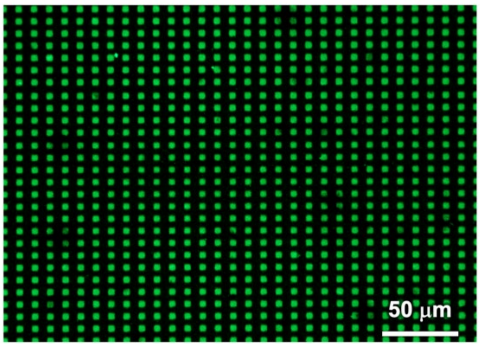

Figure S12. F-OM image of the high-resolution (~2,500 PPI) green $CsPbBr_3$ PeNC pattern obtained by the direct photocatalytic patterning process with DDT.

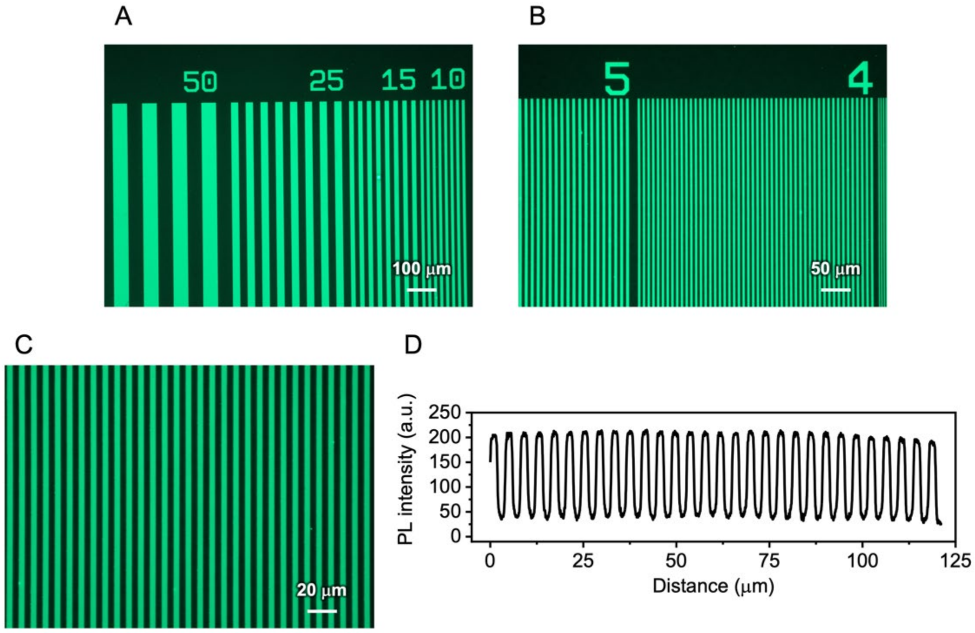

Figure S13. (A-C) F-OM images of the green $CsPbBr_3$ PeNC line pattern obtained by the direct photocatalytic patterning process with ODT. **(D)** PL intensity profile of (C).

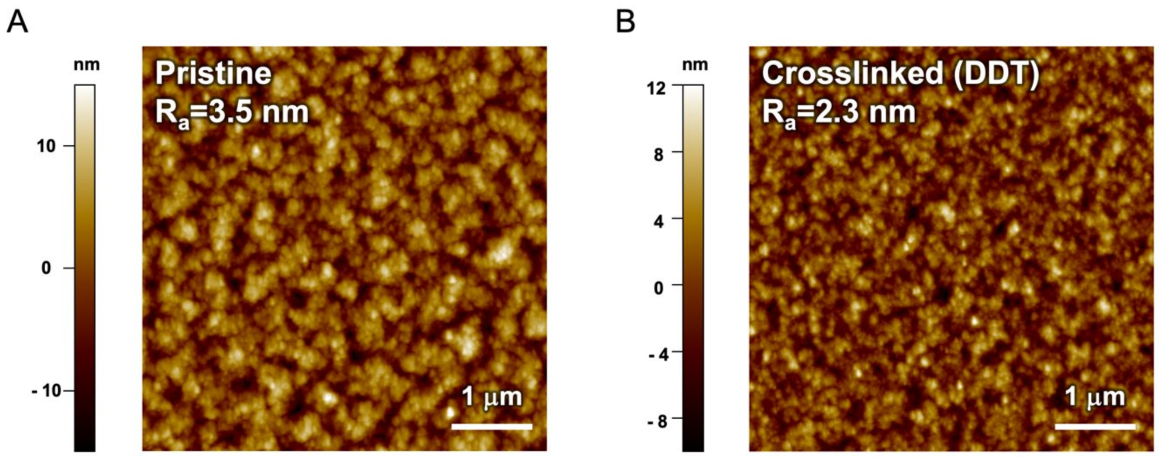

Figure S14. Atomic force microscopy (AFM) images of **(A)** pristine green $CsPbBr_3$ PeNC film and **(B)** crosslinked green $CsPbBr_3$ PeNC film with DDT. The roughness average (R_a) values for the green $CsPbBr_3$ PeNC film and crosslinked green $CsPbBr_3$ PeNC film are 3.5 nm and 2.3 nm, respectively.

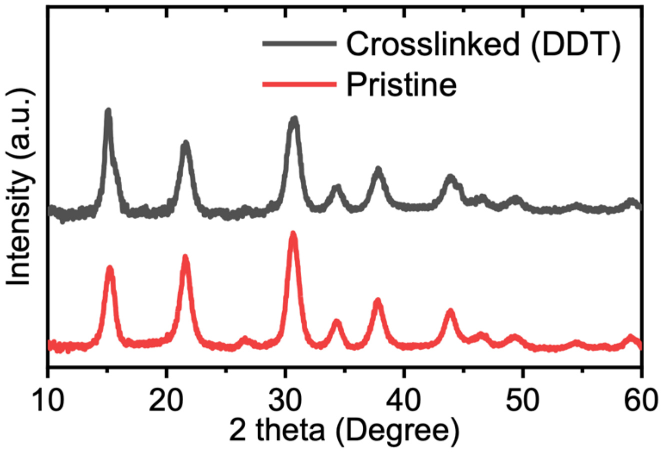

Figure S15. X-ray diffraction (XRD) patterns of green $CsPbBr_3$ PeNC film (red) and crosslinked $CsPbBr_3$ PeNC film obtained by direct photocatalytic patterning with DDT (grey).

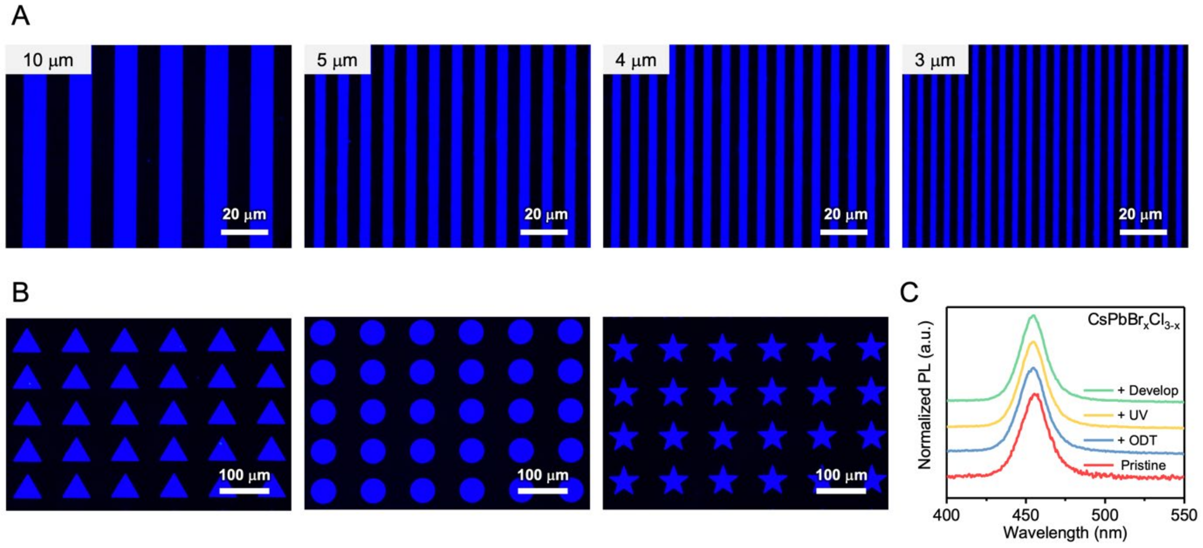

Figure S16. (A) F-OM images of the high-resolution blue $CsPbBr_xCl_{3-x}$ PeNC line pattern obtained by the direct photocatalytic patterning process with ODT. **(B)** F-OM images of triangle, dot, and star-shaped array patterns obtained by the direct photocatalytic patterning process with ODT. **(C)** PL spectra of a pristine blue $CsPbBr_xCl_{3-x}$ PeNC film and a PeNC–ODT film in each patterning step.

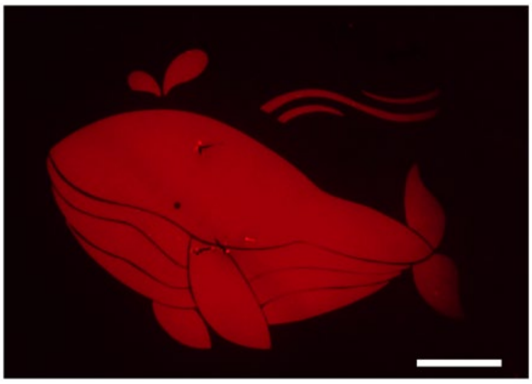

Figure S17. F-OM image of the red core–shell InP QD whale pattern obtained by the direct photocatalytic patterning process with ODT. Scale bar represents 100 μm.

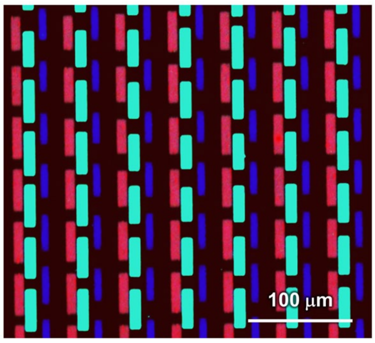

Figure S18. F-OM image of the RGB pattern. The red patterns consist of InP-based QDs, while the green and blue patterns are composed of PeNCs.

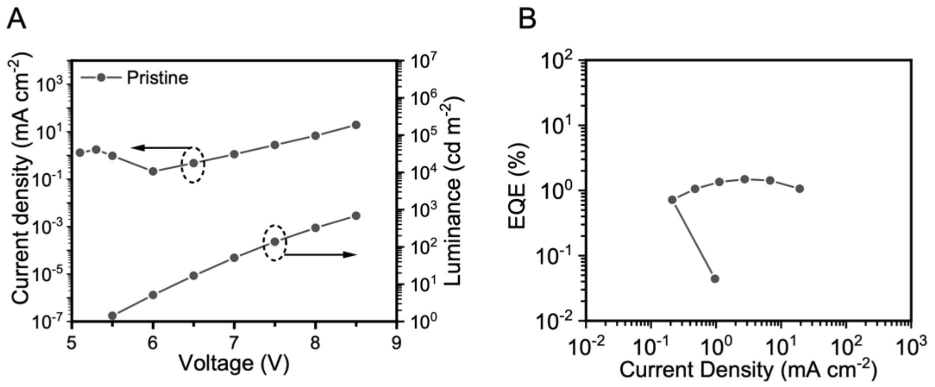

Figure S19. (A) Current density–voltage and luminance–voltage characteristics and **(B)** EQE–current density characteristics of pristine green $CsPbBr_3$ PeNC LEDs.

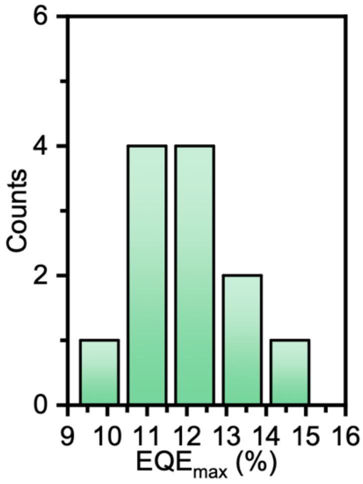

Figure S20. EQE$_{max}$ distribution for FLE-applied crosslinked PeNC LEDs using ODT crosslinker (LC (ODT) + FLE) PeNC film (N = 12).

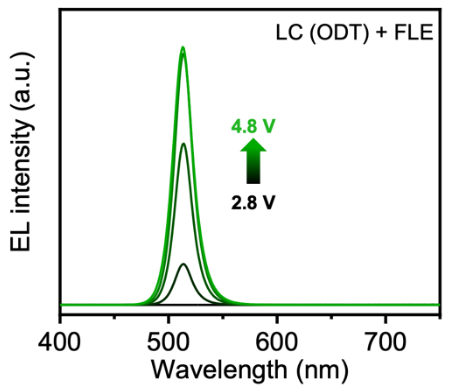

Figure S21. EL spectra of the FLE-applied crosslinked PeNC film with ODT crosslinker (LC (ODT) + FLE) under different voltage levels.

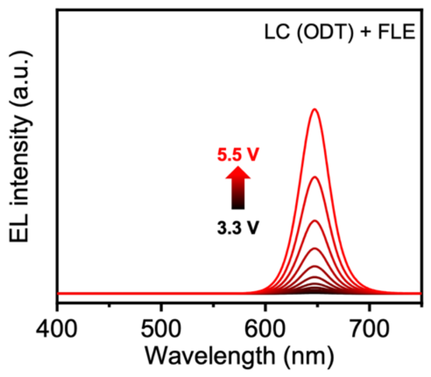

Figure S22. EL spectra of the FLE-applied crosslinked PeNC film with ODT crosslinker (LC (ODT) + FLE) under different voltage levels.

Table S1. Fitting parameters of the time-resolved PL of pristine PeNC film, PeNC film crosslinked via direct photocatalytic patterning (LC only), and FLE-applied crosslinked PeNC film (LC + FLE). ODT was used as the thiol crosslinker for direct photocatalytic patterning.

	f_1 (%)	t_1 (ns)	f_2 (%)	t_2 (ns)	t_{avg} (ns)	R^2
Pristine PeNC	82.7	2.85	17.3	16.9	5.3	0.999
LC only (ODT)	80.2	3.52	19.8	19.7	6.73	0.999
LC (ODT) + FLE	77.6	3.4	22.4	21.79	7.51	0.998

Table S2. Fitting parameters of the time-resolved PL of pristine PeNC film, PeNC film crosslinked via direct photocatalytic patterning (LC only), and FLE-applied crosslinked PeNC film (LC + FLE). DDT was used as the thiol crosslinker for direct photocatalytic patterning.

	f_1 (%)	t_1 (ns)	f_2 (%)	t_2 (ns)	t_{avg} (ns)	R^2
Pristine PeNC	85.8	2.85	14.2	17.88	4.99	0.999
LC only (DDT)	79.8	4.2	20.2	19.5	7.29	0.998
LC (DDT) + FLE	62.6	4.86	37.4	16.19	9.1	0.999

Table S3. Performance summary of $CsPbBr_3$ PeNC-LED devices under various conditions. The turn-on voltage was estimated by extrapolating the voltage-luminance curve to the point where the luminance reaches 1 cd m^{-2}.

	EQE_{max} (%)	L_{max} (cd m^{-2})	Turn-on voltage (V)
Pristine	1.49	686	5.5
FLE only	10.4	26,925	2.7
LC (ODT) + FLE (G)	14.7	13,157	2.8
LC (DDT) + FLE (G)	12.7	25,405	2.7

Table S4. Performance summary of $CsPbBr_3$ PeNC-LED devices fabricated via direct optical patterning.

**Ref.**	EQE_{max} **(%)**	L_{max} **(cd m^{-2})**
This work	14.7 (G)<br>13.1 (R)	~25,400 (G)<br>637 (R)
[25]	1.8 (G)	1,929 (G)
[37]	2.2 (G)	~ 450 (G)
[42]	0.4 (G)	105 (G)
[43]	-	1,628 (G)

References

[1] J. I. Kwon, G. Park, G. H. Lee, J. H. Jang, N. J. Sung, S. Y. Kim, J. Yoo, K. Lee, H. Ma, M. Karl, T. J. Shin, M. H. Song, J. Yang, M. K. Choi, "Ultrahigh-resolution full-color perovskite nanocrystal patterning for ultrathin skin-attachable displays", *Sci Adv* **2022**, 8, eadd0697.

[2] S. Li, Z. Wang, Y. Li, C. Su, Y. Fu, Y. Wang, X. Lu, "Fostering the Dense Packing of Halide Perovskite Quantum Dots through Binary-Disperse Mixing", *ACS Nano* **2023**, 17, 20634-20642

[3] L. Martínez, R. Andrade, E. G. Birgin, J. M. Martínez, " PACKMOL: A package for building initial configurations for molecular dynamics simulations", *J Comput Chem* **2009**, 30, 2157

[4] S. Kim, J. Chen, T. Cheng, A. Gindulyte, J. He, S. He, Q. Li, B. A. Shoemaker, P. A. Thiessen, B. Yu, L. Zaslavsky, J. Zhang, E. E. Bolton, " PubChem 2025 update", *Nucleic Acids Res* **2025**, 53, D1517

[5] A. P. Thompson, H. M. Aktulga, R. Berger, D. S. Bolintineanu, W. M. Brown, P. S. Crozier, P. J. in 't Veld, A. Kohlmeyer, S. G. Moore, T. D. Nguyen, R. Shan, M. J. Stevens, J. Tranchida, C. Trott, S. J. Plimpton, " LAMMPS - a flexible simulation tool for particle-based materials modeling at the atomic, meso, and continuum scales", *Comput Phys Commun* **2022**, 271, 108171

[6] J. Wang, R. M. Wolf, J. W. Caldwell, P. A. Kollman, D. A. Case, " Development and testing of a general amber force field", *J Comput Chem* **2004,** 25, 1157

[7] J. Park, W. Lee, J. Kim, "Large-Scale Construction and Analysis of Amorphous Porous Polymer Network Materials", *ACS Appl Mater Interfaces* **2024**, 16, 57190.

[8] W. Lee, S. Chong, J. Kim, " Graph-Network-Based Predictive Modeling for Highly Cross-Linked Polymer Systems", *arXiv* **2023**, DOI: 10.48550/arXiv.2401.06152

[9] S. Maeng, S. J. Park, J. Lee, H. Lee, J. Choi, J. K. Kang, H. Cho, " Direct photocatalytic patterning of colloidal emissive nanomaterials", *Sci Adv* **2023**, 9, eadi6950
